# An actin‐depolymerizing factor from the halophyte smooth cordgrass, *Spartina alterniflora* (*SaADF2*), is superior to its rice homolog (*OsADF2*) in conferring drought and salt tolerance when constitutively overexpressed in rice

**DOI:** 10.1111/pbi.12957

**Published:** 2018-06-28

**Authors:** Sonali Sengupta, Venkata Mangu, Luis Sanchez, Renesh Bedre, Rohit Joshi, Kanniah Rajasekaran, Niranjan Baisakh

**Affiliations:** ^1^ School of Plant Environmental and Soil Sciences Louisiana State University Agricultural Center Baton Rouge LA USA; ^2^ Southern Regional Research Center USDA‐ARS New Orleans LA USA; ^3^ Present address: Department of Biochemistry School of Dental Medicine University of Pennsylvania Philadelphia PA USA; ^4^ Present address: Escuela Superior Politécnica del Litoral Centro de Investigaciones Biotecnológicas del Ecuador Guayaquil Ecuador; ^5^ Present address: Texas A&M AgriLife Research and Extension Center Weslaco TX USA; ^6^ Present address: School of Life Sciences Jawaharlal Nehru University New Delhi India

**Keywords:** actin‐depolymerizing factor, drought, halophyte, *Oryza sativa*, salinity, *Spartina alterniflora*

## Abstract

Actin‐depolymerizing factors (ADFs) maintain the cellular actin network dynamics by regulating severing and disassembly of actin filaments in response to environmental cues. An *ADF* isolated from a monocot halophyte, *Spartina alterniflora* (*SaADF2*), imparted significantly higher level of drought and salinity tolerance when expressed in rice than its rice homologue *OsADF2*. SaADF2 differs from OsADF2 by a few amino acid residues, including a substitution in the regulatory phosphorylation site serine‐6, which accounted for its weak interaction with OsCDPK6 (calcium‐dependent protein kinase), thus resulting in an increased efficacy of SaADF2 and enhanced cellular actin dynamics. SaADF2 overexpression preserved the actin filament organization better in rice protoplasts under desiccation stress. The predicted tertiary structure of SaADF2 showed a longer F‐loop than OsADF2 that could have contributed to higher actin‐binding affinity and rapid F‐actin depolymerization in vitro by SaADF2. Rice transgenics constitutively overexpressing *SaADF2* (*SaADF2*‐OE) showed better growth, relative water content, and photosynthetic and agronomic yield under drought conditions than wild‐type (WT) and *OsADF2* overexpressers (*OsADF* 2‐OE). *SaADF2*‐OE preserved intact grana structure after prolonged drought stress, whereas WT and *OsADF2*‐OE presented highly damaged and disorganized grana stacking. The possible role of ADF2 in transactivation was hypothesized from the comparative transcriptome analyses, which showed significant differential expression of stress‐related genes including interacting partners of ADF2 in overexpressers. Identification of a complex, differential interactome decorating or regulating stress‐modulated cytoskeleton driven by ADF isoforms will lead us to key pathways that could be potential target for genome engineering to improve abiotic stress tolerance in agricultural crops.

## Introduction

The cytoskeleton is one of the most dynamic cellular components, which modulates its architecture by responding constantly to various environmental stimuli. In plants, cytoskeleton dynamics is critical for numerous cellular processes, such as cell division, morphogenesis, polarized cell expansion, root and pollen tube tip growth, cytoplasmic streaming/cyclosis (Menand *et al*., 2007; Pollard and Cooper, 2009), cell‐to‐cell communication through plasmodesmata (Higaki *et al*., 2008), perception of gravitropism (Kordyum *et al*., 2009; Stanga *et al*., 2009), regulation of cell shape (Smith and Oppenheimer, 2005), and in response to wounding, pathogen attack, hormone distribution and cold acclimation (Deng *et al*., 2010; Hussey *et al*., 2006; Staiger, 2000; Staiger and Blanchoin, 2006; Wasteneys and Galway, 2003).

Filamentous actin (F‐actin) network constitutes majority of the cytoskeleton (Li *et al*., 2015). The stochastic dynamics of the F‐actin via polymerization, depolymerization, severing, nucleation and large‐scale cellular translocation events affect the overall cytoskeletal integrity (Augustine *et al*., 2011). Actin remodelling plays an important role in plant cell, tissue, and organ development reprogramming, cell division and cellular organelles assembly. Actin also predictably participates in nucleosome occupancy, chromatin modification and regulation of gene expression (Bettinger *et al*., 2004; Miralles and Visa, 2006). Actin, in coordination with a large group (over 70 families) of both cytoplasmic and nuclear actin‐binding proteins (ABPs), provides the cytoskeleton with high plasticity during growth and environmental challenges (Augustine *et al*., 2011; Deng *et al*., 2010; Tholl *et al*., 2011). ABPs, singly or in combination, regulate the stoichiometric ratio between the free monomeric G‐actin (globular actin) and constantly depolymerizing F‐actin in plant cell. Of the total pool of actin moieties, only 5% usually remains in filamentous state at a given time (Gibbon *et al*., 1999; Snowman *et al*., 2002). The constant entry and exit of the G‐actin pool within the cytoskeletal mesh requires a number of ABPs and their functional partners to be expressed and active during the process.

Actin‐depolymerizing factors (ADFs)/cofilins are a family of ubiquitous, low molecular mass (15 to 20 kDa) ABPs that bind both the G‐actin and F‐actin in plants, and their functions are regulated by cellular pH, ionic strength and the availability of other binding partners (Li *et al*., 2010). ADF is reportedly essential for plant viability (Augustine *et al*., 2008). By binding to the ADP‐bound form of actin, ADFs sever actin filaments and thus provide more barbed filament ends for polymerization (Clément *et al*., 2009; Li *et al*., 2010; Staiger *et al*., 2009; Tian *et al*., 2009). ADFs also increase the rate of dissociation of F‐actin monomers from the pointed ends by changing the helical twist of the actin filament, thus accelerating the dissociation of subunits (Bamburg and Bernstein, 2008; Bowman *et al*., 2000; Cooper and Schafer, 2000; Daher *et al*., 2011). These two activities together make ADFs to be the major regulator of actin dynamics in plant cell, with important functional association with other regulatory proteins, for example actin‐interacting protein 1 (AIP1, Amberg *et al*., 1995; Iida and Yahara, 1999; Konzok *et al*., 1999) and calcium‐dependent protein kinase (CDPK, Smertenko *et al*., 1998).

To date, only a few plant ADFs, such as *Arabidopsis* (Bowman *et al*., 2000; Carlier *et al*., 1997; Nan *et al*., 2017; Tholl *et al*., 2011), maize *ZmADF* (Gungabissoon *et al*., 1998) and a pollen‐specific ADF from lily (Allwood *et al*., 2002), have been biochemically characterized. The inhibition of ADF activity by phosphatidylinositol 4, 5‐bisphosphate and phosphatidylinositol 4‐monophosphate and that they can also shut down phospholipase C activity reveal a close association of ADFs with phosphoinositide signalling in plants (Gungabissoon *et al*., 1998). Phosphorylation of plant ADFs at the conserved serine‐6 residue by CDPK inhibits their depolymerization activity (Allwood *et al*., 2001; Smertenko *et al*., 1998), which suggests that Ca^2+^ status of the cell may play an important role in the regulation of ADF activity.

Drought and salinity are the two most important environmental stressors that negatively impact the growth and productivity of agricultural crops, including rice, arguably the most important global food crop. Plants, as sessile organisms, have developed strategies to adapt to these stresses by physiological and biochemical adjustments achieved through the coordinated expression of genes involved in stress‐responsive gene regulatory networks. Many ABPs influence actin filament dynamics in response to environmental signals (Hussey *et al*., 2006; McCurdy *et al*., 2001; Staiger and Blanchoin, 2006; Yokota and Shimmen, 2006). Plant cytoskeleton is thus emerging as an active receiver of environmental stress signals through the recruitment of ABPs, including ADFs (Drobak *et al*., 2004; Solanke and Sharma, 2008). However, there are only a few reports of implications of ADFs in abiotic stress response. *TaADF* was regulated specifically under cold stress in wheat (Ouellet *et al*., 2001). A hydrophobic ADF mutant (valine 69 to alanine) was shown to rescue a partial RNA interference‐mediated stunted growth phenotype at a permissive temperature (20 to 25 °C) but not at 32 °C, a restrictive temperature (Vidali *et al*., 2009) in temperature‐sensitive candidates of moss, *Physcomitrella patens*. Freezing induced an ADF activity leading to depolymerization of actin filaments in oilseed rape (Egierszdorff and Kacperska, 2001). ADF was up‐regulated in rice after 2 to 6 days of drought stress (Ali and Komatsu, 2006). Rice *OsADF3* was shown to be induced under stress and enhance drought stress tolerance in *Arabidopsis* (Huang *et al*., 2012).

Halophytes adapt to salt and drought by virtue of their superior alleles of the genes involved ion homeostasis, osmotic adjustment, ion extrusion and compartmentalization in comparison with glycophytes (Zhu, 2000). Many halophytes, such as *Thellungiella halophila* (Wu *et al*., 2012), *Mesembryanthemum crystallinum* (Chiang *et al*., 2016; Tsukagoshi *et al*., 2015), *Porteresia coarctata* (Majee *et al*., 2004), have been proved to be elite source of stress tolerance genes for bioprospecting. A perennial grass halophyte, *Spartina alterniflora* (Loisel) (smooth cordgrass), is reported to grow in salinity ranging from 5 to 32 psu, that is double the strength of marine water (Baisakh *et al*., 2008). Along with *P. coarctata*,* S. alterniflora* was proposed to be a model halophyte grass for monocotyledonous crops (Joshi *et al*., 2015; Subudhi and Baisakh, 2011). Bioprospecting of *S. alterniflora* genes has been reported to improve salinity and drought stress resistance when overexpressed in model plant *Arabidopsis* and rice (Baisakh *et al*., 2012; Joshi *et al*., 2013, 2014). The present study emanates from the hypothesis that modulation of cytoskeleton architecture by manipulating actin turnover provides abiotic stress resistance in crops and that an ADF of a halophyte is superior to its homolog from the glycophytic rice. Here, we report on the biochemical and functional implications of an ADF from *S. alterniflora* (*SaADF2*) in drought and salt stress response when overexpressed in rice. Further, its superiority over rice homolog *OsADF2* was studied by overexpressing *OsADF2*. Structural differences between the two highly identical proteins as possible reasons of the functional superiority of the former in conferring abiotic stress tolerance are discussed.

## Results

### *SaADF2* was highly identical to OsADF2 and nuclear localized

**Figure 1:**
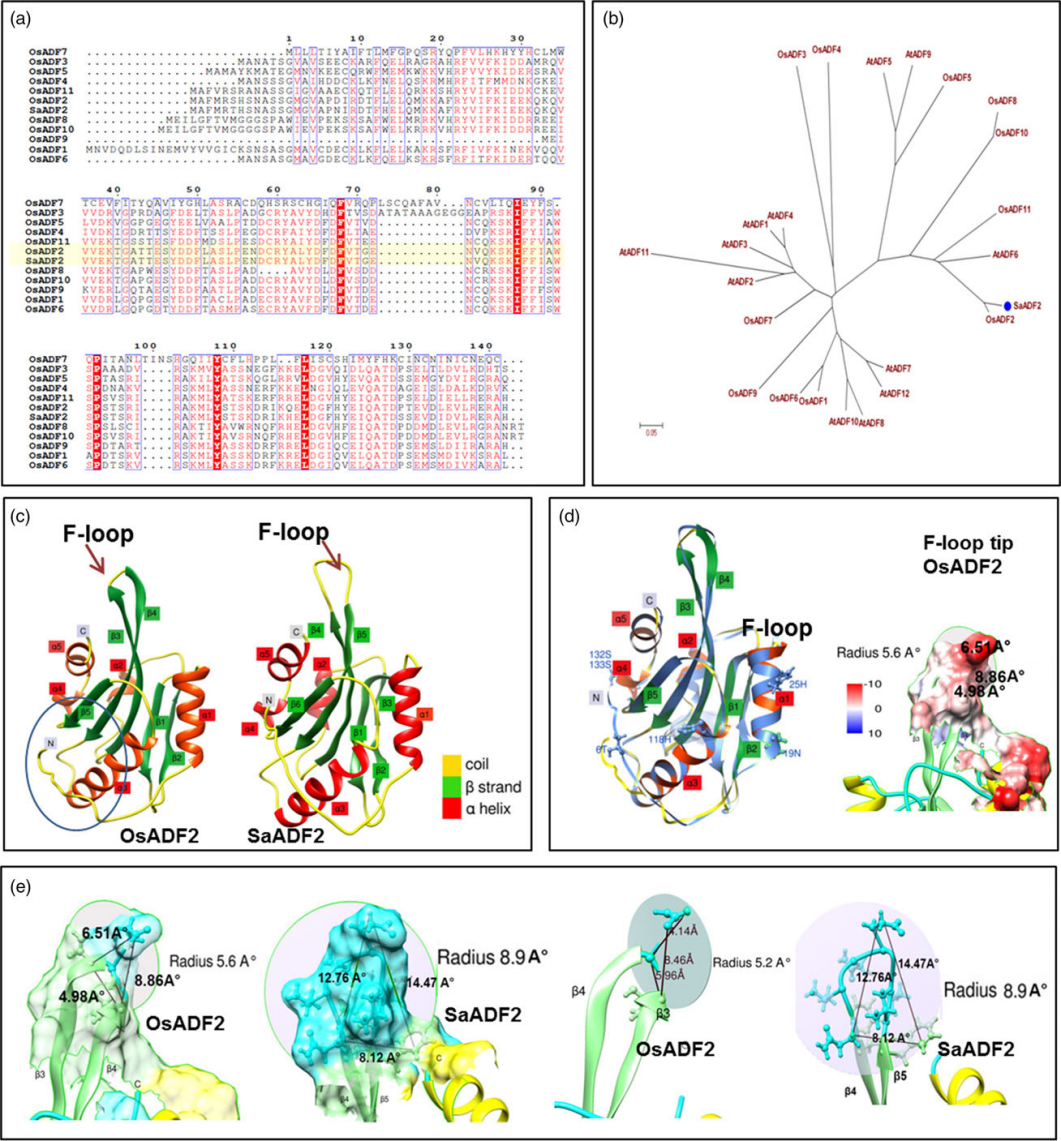
Sequence analysis of actin depolymerization factor (SaADF2) of *Spartina alterniflora*. Multiple sequence alignment of SaADF2 and rice ADFs (a). Phylogenetic relationship among SaADF2, rice and *Arabidopsis* ADFs (b). Homology modelling‐based tertiary structures of OsADF2 and SaADF2 (c). Ribbon models of OsADF2 (rainbow) and SaADF2 (blue) aligned in three‐dimensional space (right), with the amino acid differences positioned on SaADF2. The globular model for OsADF2 shows the small F‐loop tip. Red to white‐to‐blue colour transition indicates hydrophobic to hydrophilic transition of the protein surface (d). Dimension of the coil component of F‐loop in SaADF2 and OsADF2 (e).

A 438‐bp‐long cDNA isolated from abiotic stress‐responsive transcriptome of the grass halophyte *Spartina alterniflora* (Baisakh and Mangu, 2016) codes for a conserved and ubiquitous actin‐binding protein (ADF) of 145 amino acid residues. Protein sequence comparison of *S. alterniflora* ADF with ADF gene family members from rice showed that *S. alterniflora* ADF was >95% identical to rice ADF isoform, OsADF2, and hence was annotated as SaADF2 (Figure 1a), which clustered with AtADF6 from *Arabidopsis* (Figure 1b). SaADF2 showed nuclear localization (Figure S1), as was observed for OsADF2 by Huang *et al*. (2012).

### SaADF2 is structurally different from OsADF2

SaADF2 is a typical plant ADF with a highly conserved cofilin/ADF domain spanning the C‐terminus of the monomer (ADF‐H domain, N19 to H145). It weighs 16.8 kDa with a predicted pI of 6.20, while it is 5.65 kDa for OsADF2. The core structures of SaADF2 and OsADF2 are highly similar with five central α‐helices and five β‐strands, but SaADF2 shows a sixth β‐strand at the N‐terminal end (Figure 1c). Predicted tertiary structures showed a central core barrel of β4 and β5 in SaADF2 (β3 and β4 in OsADF2), surrounded by α‐helices and other β‐sheets. The two core β‐sheets are joined by the F‐loop (Figure 1d), a flexible coil responsible for F‐actin binding (Singh *et al*., 2011). In OsADF2, the F‐loop is 6.50 to 8.86 Å high from N‐ and C‐terminal side, respectively, with a base 4.98 Å and active plane radius 5.6 Å. Contrastingly, the F‐loop in SaADF2 is 12.76 and 14.47 Å high from β4 and β5 (N and C termini), respectively, with active plane radius 8.9 Å (Figure 1e). The long F‐loop of SaADF2 is significantly exposed outside the protein core providing it a high rotational free space. The F‐loop tip is highly hydrophilic and organized with two hydrophobic patches on both sides of SaADF2, whereas the hydrophilic tip volume is much reduced in OsADF2 (Figure 1d). OsADF2 and SaADF2 differ by six amino acid substitutions (Figure 1d). Serine 6, the key phosphorylation site of plant ADFs, is substituted in SaADF2 by threonine. At the helix subproximal to C‐terminus in SaADF2, phosphosensitive proline 132 and threonine 133 are both substituted by serine in OsADF2. Other substitutions, 19N>19D, 25H>25L and 118H>118Q, are positioned on OsADF2 model superimposed over SaADF2 (Figure 1d). The Mn^+^ ligand binding sites on both proteins are K106 and R102.

### SaADF2 had greater actin‐binding affinity than OsADF2 i*n vitro*

**Figure 2:**
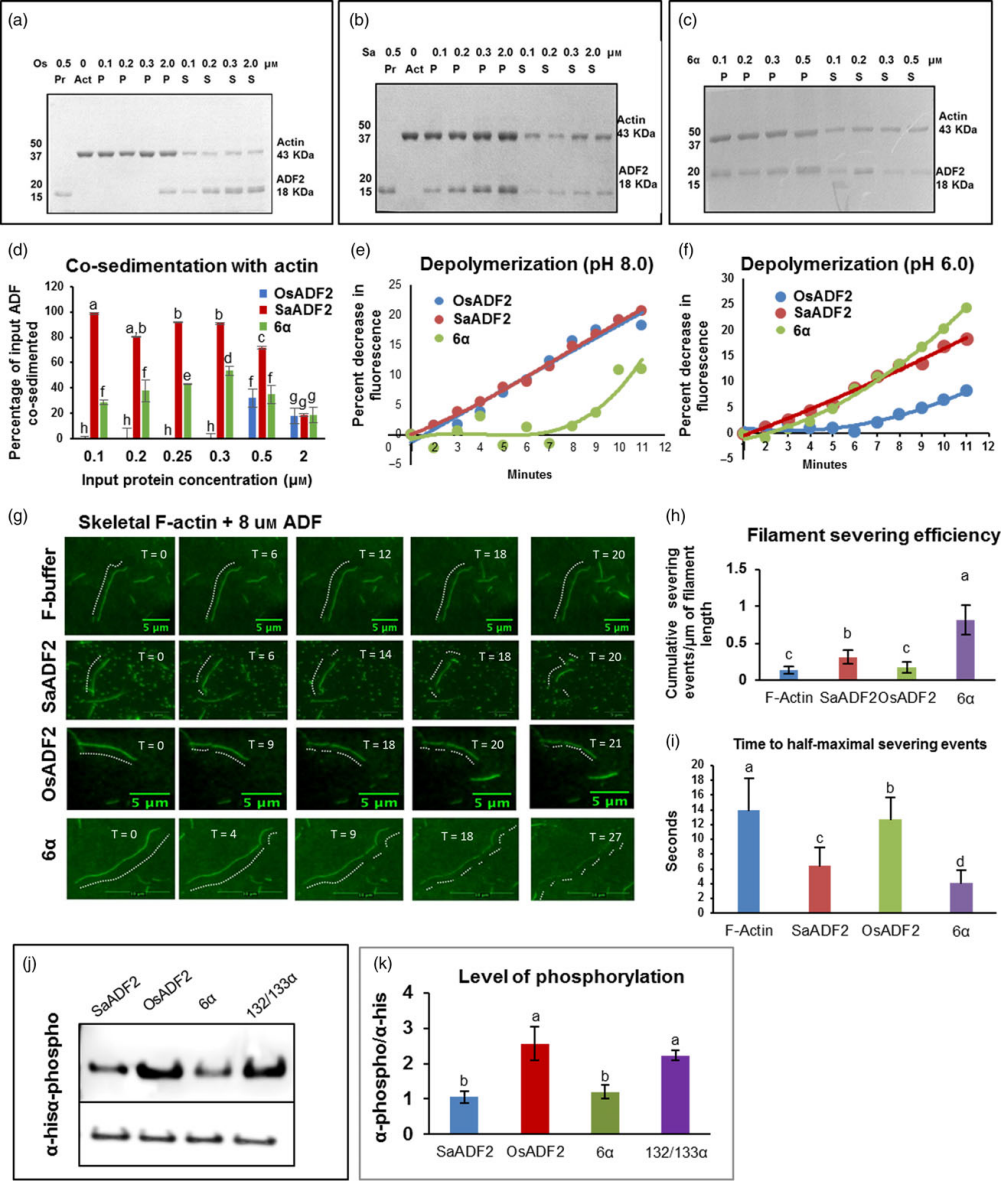
Low‐speed co‐sedimentation assay showing binding of actin bundles/G‐actin with OsADF2 (a, d, SaADF2 (b, d), and OsADF2/6α (c, d). Approximate degree of binding was measured by densitometry scanning of the gel (d). Depolymerization activity of SaADF2, OsADF2 and OsADF2/6α proteins using prepolymerized F‐actin at pH 8.0 (e) and pH 6.0 (f)). Severing and depolymerization of steady‐state actin single‐filaments incubated with 8 μm SaADF2, OsADF2 and OsADF2/6α (g‐i). Analysis of severing activities (h) and average time to half‐maximal severing (i) by the proteins of interest (POI) and in the absence of protein (F‐actin) is shown at end time point of the assay (*n* = 5). Inhibitory phosphorylation of SaADF2, OsADF2, OsADF2/6α and another phosphosensitive mutant, OsADF2/132/133α, by CDPK shown by immunoprecipitation (j) followed by densitometric quantification (k). Data are shown as means with standard error of means, *n* = 3).

Immunoblotting results showed that the recombinant SaADF2 and OsADF2 proteins (17 kDa) were mostly expressed in the membrane fraction of the prokaryotic system (Figure S2; Appendix S1). F‐actin binding and bundling assay showed that both SaADF2 and OsADF2 co‐sediment with actin at low‐speed centrifugation in a concentration‐dependent manner (Figure 2a–d). Both proteins bound to G‐actin monomer and F‐actin bundles, but their actin‐binding efficiency differed with protein concentration. In a dose‐dependent assay using 0.1 to 2 μm protein concentration, actin (3 μm) co‐sedimented with SaADF2 at 0.1 μm (Figure 2b,d), whereas OsADF2 started to co‐sediment only at 0.5 μm, and at low concentration, a major portion of the protein remained in the supernatant fraction (Figure 2a,d). Both proteins at 2 μm co‐sedimented with 20% input actin (Figure 2d). However, at 0.5 μm ADF2, the binding of SaADF2 was twofold higher than OsADF2. At 0.1 to 0.3 μm concentration range, OsADF2 showed no binding, but SaADF2 showed binding proportionate to protein concentration (Figure 2d). On the other hand, OsADF2/6α mutant protein with serine‐6 replaced by threonine had an actin‐binding efficiency equivalent to SaADF2, that is the protein co‐sedimented at its lowest concentration (0.1 μm) with actin and the amount of co‐sedimented protein increased with increase in its concentration (Figure 2c,d).

### SaADF2 depolymerized F‐actin filaments at a wider pH range and more efficiently than OsADF2

F‐actin binding as well as depolymerizing activity of the purified recombinant ADF2 proteins was monitored by a fluorescence assay by incubating the proteins with 0.8 μm pyrene‐labelled actin. Fluorescence quenching of undiluted F‐actin suggested that both ADF2s bind to F‐actin and thus promote actin depolymerization and enhance actin turnover rate (Figure 2e–h). At pH 8.0, both ADF2s showed comparable F‐actin depolymerization activity (Figure 2e). While SaADF2 showed 20% decrease in fluorescence over a 10‐min period, OsADF2 showed 18% decrease (Figure 2e). However, at pH 6.0, OsADF2 lost its potency to depolymerize F‐actin significantly and the decrease in fluorescence dropped to 9%, whereas SaADF2 maintained its depolymerization activity at 18% (Figure 2f). Interestingly, OsADF2/6α showed slow, early depolymerizing activity at pH 8.0, which increased to 12% by 11 min (Figure 2e), but at pH 6.0, it had the highest (23%) actin depolymerization activity (Figure 2f). In the absence of any binding protein in vitro, 4‐ to 8‐μm‐long actin filaments were observed by total internal reflection fluorescence (TIRF), which began dissociating following disassembly (severing/depolymerization) in the presence of excess (8 μm) of the ADF proteins (Figure 2g–i). SaADF2 predominantly severed the filaments by depolymerizing from ends, whereas OsADF2 mostly disassembled them into shorter fragments (Figure 2g). Steady‐state actin single filaments showed more severing and depolymerization by SaADF2 and OsADF2/6α as compared to slow and moderate severing by OsADF2 (Figure 2h,i). Higher depolymerization by SaADF2 may have led to more enrichment of the G‐actin pool than by OsADF2.

### OsCDPK6 preferentially phosphorylated OsADF2 at serine‐6

Immunoblot with antiphosphoserine antibody showed apparent difference in the degree of phosphorylation by the ADF2s (Figure 2j,k). OsADF2 produced at least two times higher phosphorylation signal compared to SaADF2 and OsADF2/6α (Figure 2k). Although threonine is also phosphorylated by the promiscuous CDPK, its preference for serine‐6 was evident with no substantial change in phosphorylation of the OsADF2 mutated at two other serine sites, 132 and 133 (Figure 2j,k).

### *SaADF2* and *OsADF2* overexpression conferred contrasting drought tolerance phenotypes

**Figure 3:**
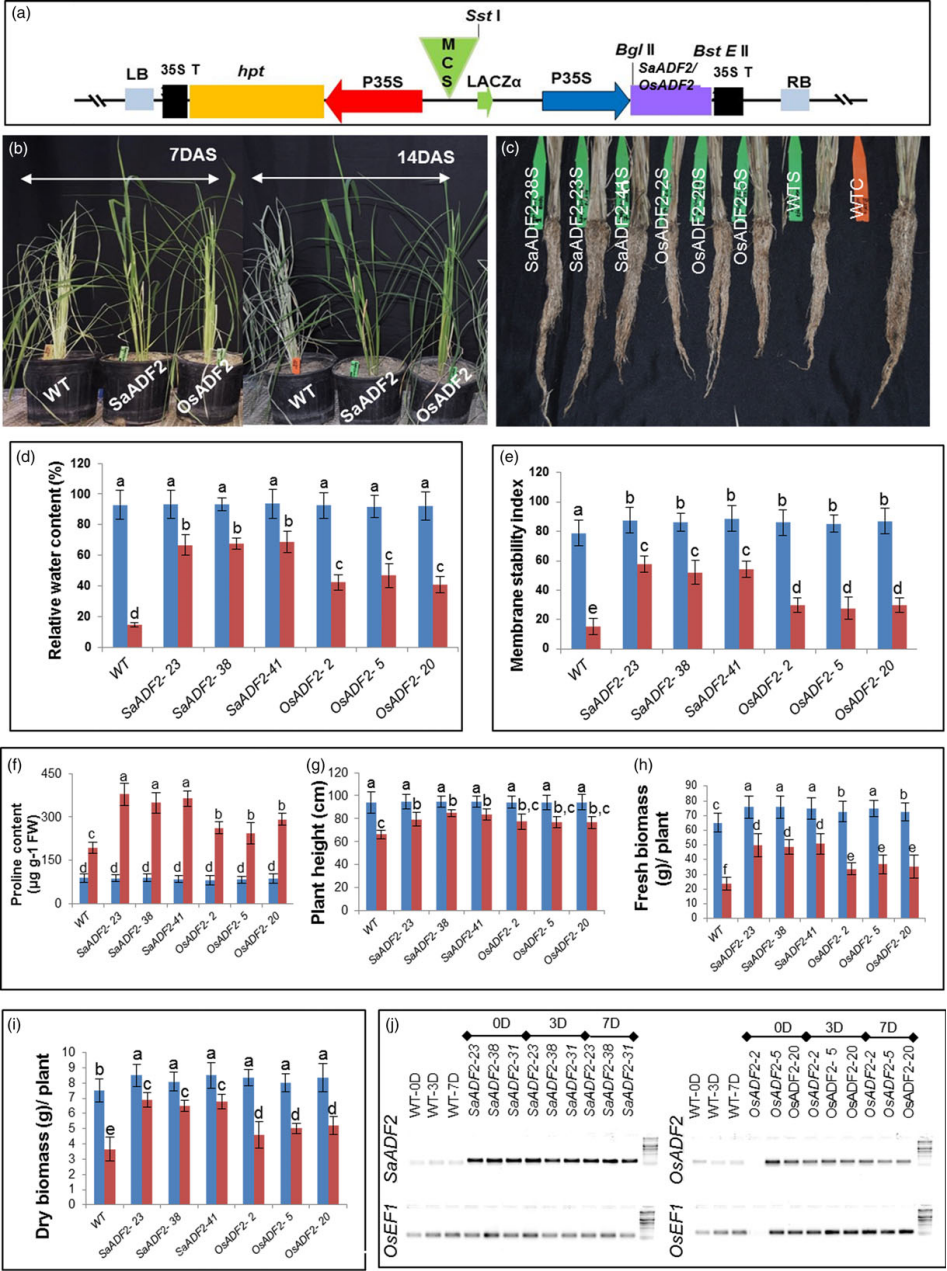
T‐DNA structure of rice transformation vector p35S:SaADF2 containing expression cassettes of *SaADF2*/*OsADF2* and hygromycin phosphotransferase (*hpt*) under the control of CaMV 35S promoter (P35S). MCS = multicloning site, LB = left border, RB = right border, 35S T = CaMV 35S terminator (a). Shoot (b) and root morphology (c) and physiological traits of transgenic *SaADF2*/*OsADF2* overexpressers (OE) plants after 7 days of drought stress (DAS): % relative water content (d), membrane stability index (e), proline content (f), plant height (g), fresh biomass (h) and dry weight (i). Blue and red bars represent control (unstressed) and stressed conditions, respectively. Expression of *SaADF2* and *OsADF2* under control (0D), and 3 days (3D) and 7 days (7D) after drought stress (7D) (j). The faint nonspecific signals for *SaADF2* in WT are from the endogenous *OsADF2*. Data are presented as means with standard error of means (*n* = 3). Bars topped with different letters represent values that are significantly different (*P* < 0.05) at 0 day or 7 day after stress.

Thirty‐two and 15 primary transgenic rice ‘Nipponbare’ lines overexpressing *SaADF2* and *OsADF2*, respectively, under the control of the constitutive cauliflower mosaic virus 35S promoter (Figure 3a) (hereinafter referred to as *SaADF2*‐OE and *OsADF2*‐OE) were generated. T_1_ lines showing single‐copy Mendelian inheritance and expression of *ADF* genes were grown for attainment of homozygosity and further analysis.

Under 7–14 days after drought stress (DAS) at prebooting stage (10% FC), the *SaADF2*‐OE showed significantly greater tolerance with less wilting, withering, and reduction in shoot and root growth than the *OsADF2*‐OE and WT plants (Figure 3b,c,g–i). Upon resuming irrigation, *SaADF2‐OE* recovered to normal growth quickly as compared to the WT (Figure S3; Appendix S1). Under well‐watered control condition, no significant difference was observed in growth and development between WT and *SaADF2*‐OE (Figure S3).

### *SaADF2*‐OE held high relative water content and stomatal conductance under drought stress

**Figure 4:**
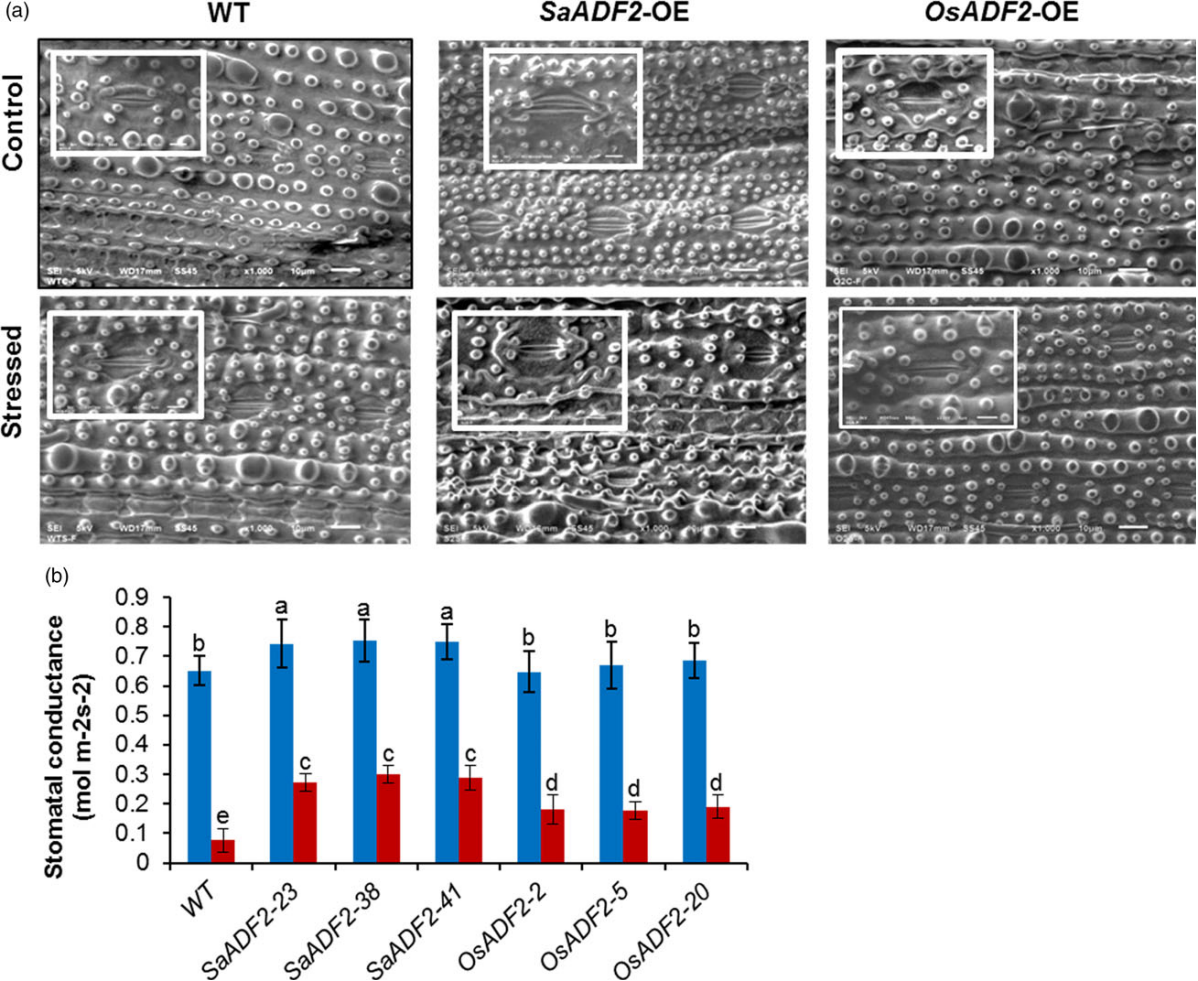
Scanning electron micrographs showing stomatal aperture (a, b), and stomatal conductance (c) of overexpressers of *SaADF2* and *OSADF2* vis‐à‐vis WT 7d after drought. Data are presented as means with standard error of means (*n* = 3). Blue and red bars represent control (unstressed) and stressed conditions, respectively. Bars topped with different letters represent values that are significantly different (*P* < 0.05) at 0 day or 7 day after stress.

Relative water content (RWC), the physiological ability of a cell to maintain water status through osmotic adjustment, was ~90% for both WT and transgenic plants. However, RWC of WT and *OsADF2*‐OE plants dropped to 15% and 43%–45% 7 DAS. On the other hand, *SaADF2*‐OE maintained 65%–70% RWC 7 DAS (Figure 3d). *SaADF2*‐OE maintained higher membrane stability (Figure 3e), accumulated more proline (Figure 3f) under drought as compared to *OsADF2*‐OE and WT. Similar observations were recorded for other agronomic traits, such as plant height (Figure 3g), and fresh and dry plant biomass (Figure 3h,i), where *SaADF2*‐OE maintained superiority over WT and *OsADF2*‐OE following drought. The expression of *SaADF2* was maintained under drought while *OsADF2* expression reduced under drought (Figure 3j). Scanning electron micrographs revealed that *SaADF2*‐OE maintained stomatal aperture opening comparable to control condition, but *OsADF2*‐OE showed reduced aperture opening and WT showed complete closure of stomatal aperture with visibly shrunken guard cells under drought (Figure 4a). *SaADF2*‐OE efficiently maintained the cellular osmotic potential under water deficit with less reduction in stomatal conductance than *OsADF2*‐OE and WT (Figure 4b).

### *SaADF2*‐OE lines maintained intact chloroplast ultrastructure, higher chlorophyll content and photosynthesis under drought stress

**Figure 5:**
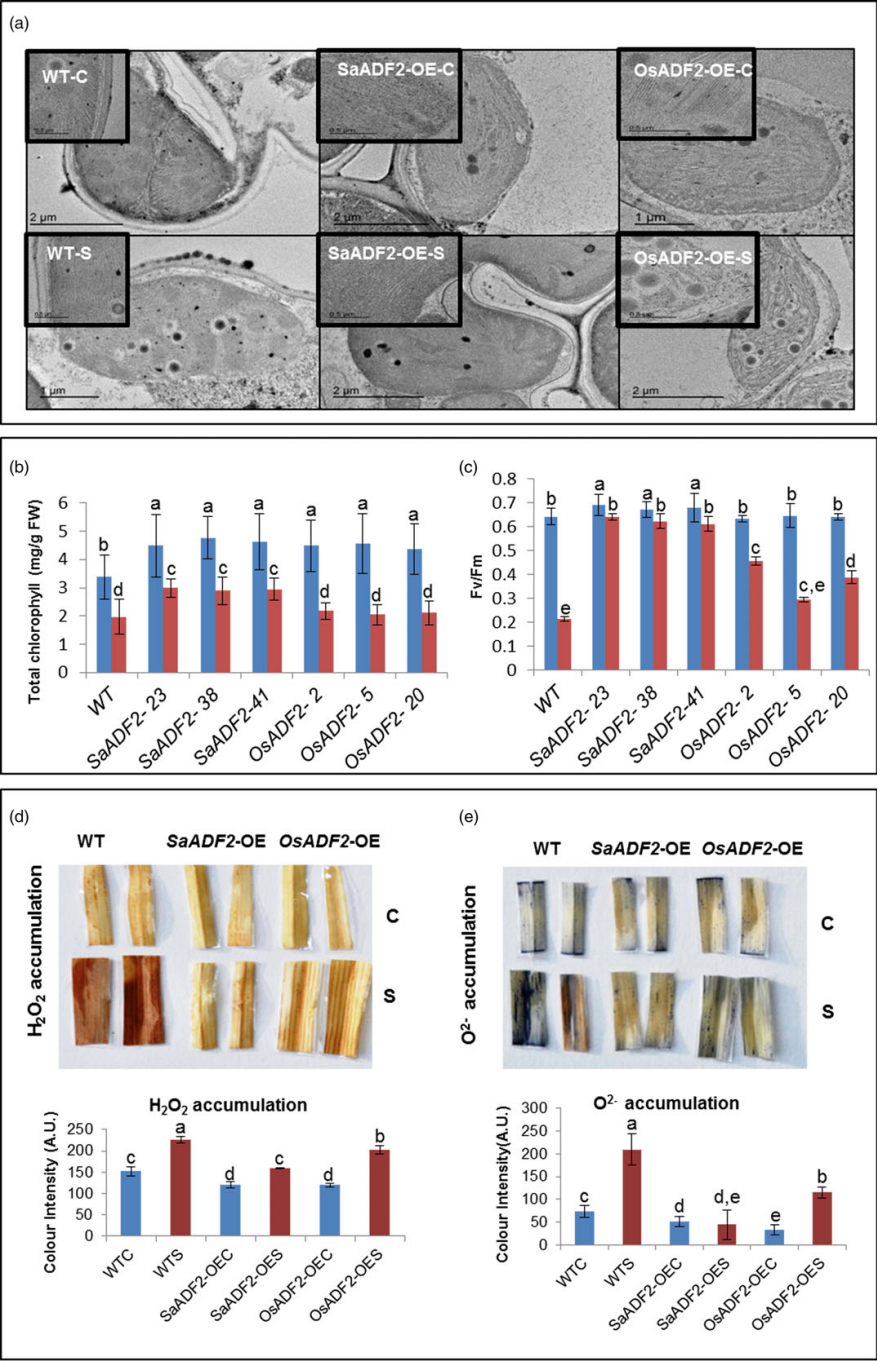
Transmission electron micrographs showing chloroplast ultrastructure with thylakoid membrane arrangement of overexpressers of *SaADF2* and *OSADF2,* and WT under control C) and drought stress (S) (a). Total chlorophyll content (b), photosynthetic performance (Fv/Fm) (c), DAB assay showing H_2_O_2_ (d) and NBT assay showing O^2−^ (e) accumulation in overexpressers of *SaADF2* and *OsADF2* vis‐à‐vis WT under control (C) and drought stress (S). Blue and red bars represent control (unstressed) and stressed conditions, respectively. Data are presented as means with standard error of means (*n* = 3). Bars topped with different letters represent values that are significantly different (*P* < 0.05) at 0 day or 7 day after stress.

Control plants showed well‐defined, normal kidney‐shaped chloroplasts with clearly distinct envelope membranes and a well‐developed internal membrane system with evenly distributed, well‐packed grana and long stromal thylakoids (Figure 5a). Drought caused disintegration of the chloroplast fine structures including outer membrane and thylakoid disorganization with disoriented grana stacking; many plastoglobuli appeared with high electron density in both WT and *OsADF2*‐OE (Figure 5a). In contrast, typical fine structure of chloroplasts was conserved in *SaADF2*‐OE at 7 DAS. Chloroplastids were disarranged in WT plant mesophyll cells as compared to the regular arrangement in *SaADF2*‐OE (Figure S3, Appendix S1).

*SaADF2*‐OE maintained higher chlorophyll concentration than *OsADF2*‐OE and WT at 7 DAS (Figure 5b). *SaADF2*‐OE showed better photosynthetic performance as reflected by less damage to photosystem II with higher Fv/Fm over *OsADF2*‐OE and WT under drought stress (Figure 5c), which indicated that *SaADF2*‐OE plants were less sensitive to drought‐induced photo‐inhibition.

### *SaADF2*‐OE produced less reactive oxygen species (ROS) than *OsADF2*‐OE and WT plants under drought stress

Both *SaADF2*‐OE and *OsADF2*‐OE accumulated less ROS compared to WT under drought as shown by less coloration of the leaves in DAB and NBT assay. DAB assay showed the accumulation of H_2_O_2_ in only mid‐vein region of *SaDAF2*‐OE (Figure 5d). On the contrary, *OsADF2*‐OE demonstrated higher accumulation of ROS with more coloration in the vein and H_2_O_2_ accumulation all over the leaf strip (Figure 5d). *SaADF2*‐OE lines were particularly superior with very less characteristic dark blue coloration of the leaf strips in NBT assay (Figure 5e). O^2−^ accumulation in the leaves of *SaADF2*‐OE under drought was comparable to control condition.

### *SaADF2*‐OE were agronomically superior under drought stress

**Figure 6:**
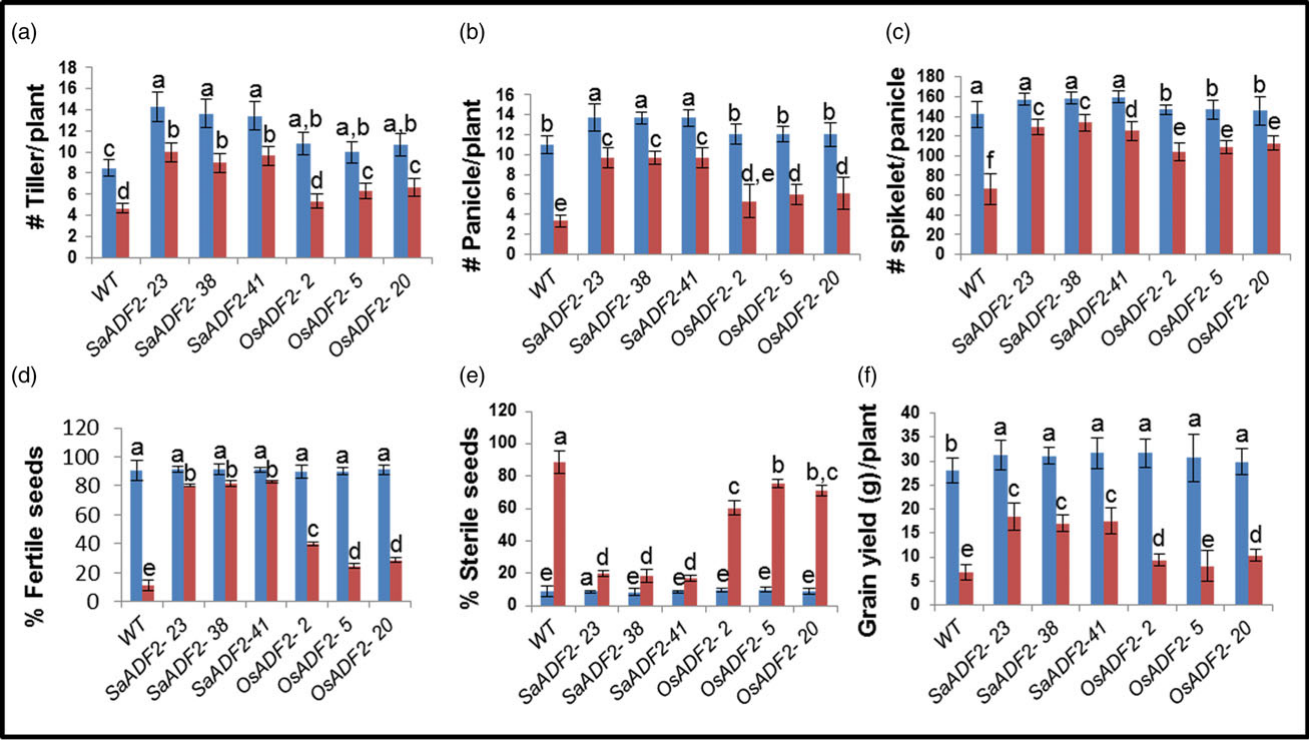
Postharvest yield parameters (a–f) and other agronomic traits (g–i) of 1‐week drought‐stressed overexpressers of *SaADF2* and *OsADF2* vis‐à‐vis WT. Data are presented as means with standard error of means (*n* = 3). Blue and red bars represent control (unstressed) and stressed conditions, respectively. Bars topped with different letters represent values that are significantly different (*P* < 0.05) at 0 day or 7 day after stress.

Drought‐stressed *SaADF2*‐OE had markedly higher grain yield and yield attributing traits compared to *OsADF2*‐OE and WT (Figure 6). Drought‐stressed *OsADF2*‐OE and WT showed ~45% and ~41% reduction in tiller number compared to 28% in *SaADF2*‐OE (Figure 6a). There was ~70% and ~50% decrease in panicle number in WT and *OsADF2*‐OE compared to 30% in *SaADF2*‐OE (Figure 6b). Panicles had less spikelets in *OsADF2*‐OE than WT and *SaADF2*‐OE under unstressed condition, and drought caused a reduction of 53%, 26%, and 18% in WT, *OsADF2*‐OE and *SaADF2*‐OE, respectively (Figure 6c). A striking difference was noted in the fertile seeds count, which declined by about 88% in WT, 66% in *OsADF2*‐OE, but only 11% in *SaADF2*‐OE (Figure 6d,e). This was reflected in grain yield per panicle where drought caused 76% and 70% yield reduction in WT and *OsADF2*‐OE compared to only 31% in *SaADF2*‐OE (Figure 6f). Interestingly, *ADF2*‐OE showed some superiority over the WT plants for reproductive traits, such as number of tillers and panicles per plant, and spikelet number (Figure 6a–c) as well as vegetative growth traits, such as fresh and dry biomass (Figure 3h,i) under control conditions. This suggested that ADF2 overexpression conferred an overall growth benefit to the transgenic plants.

### *SaADF2* overexpression conferred salt tolerance in rice plants

**Figure 7:**
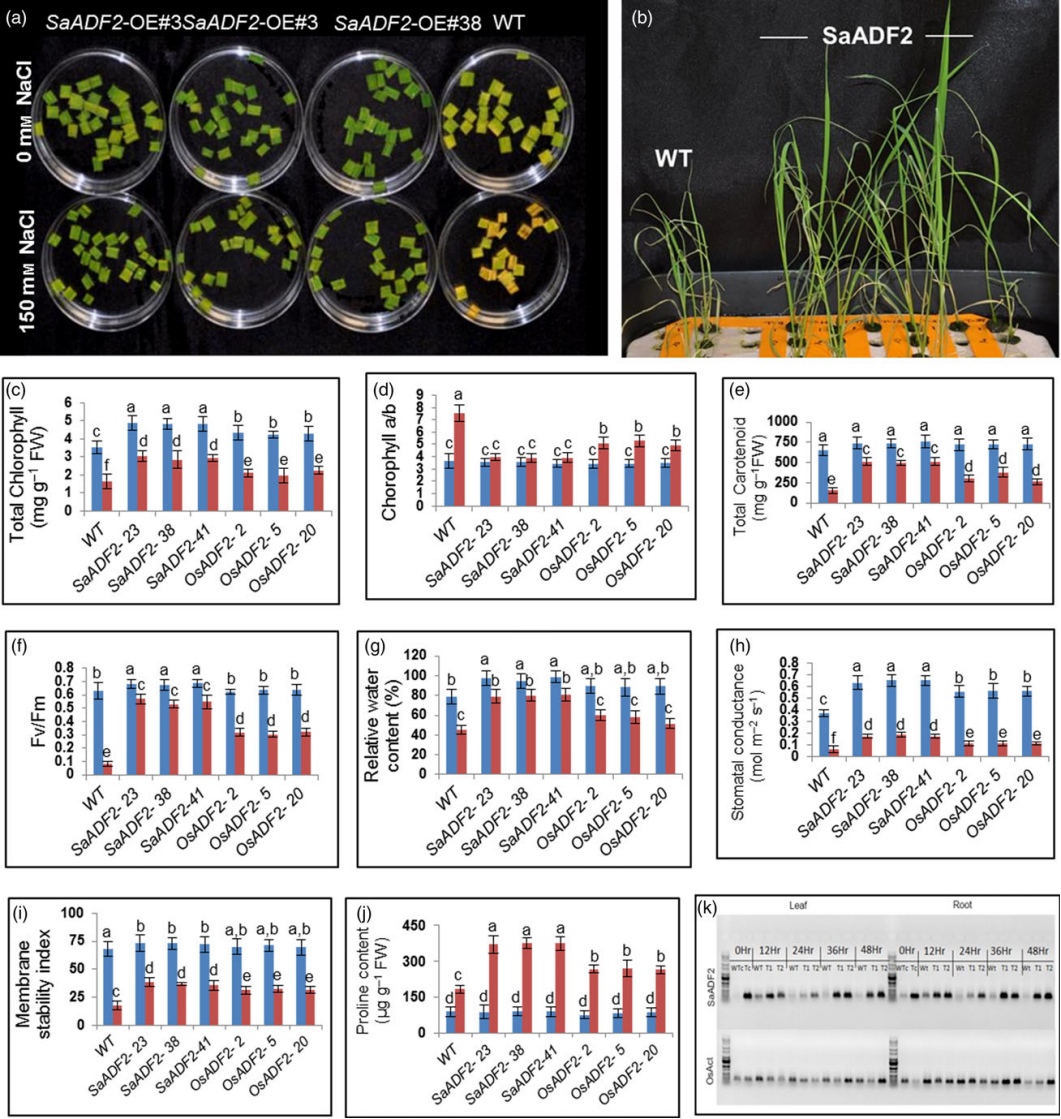
Salt tolerance assay showing greater tolerance of *SaADF2*‐overexpressing transgenics compared to WT. Cut‐leaf float assay (a), seedling assay (b) and physiological parameters (c‐j). Temporal accumulation of *SaADF2* transcript in leaf and root tissue of *SaADF2*‐overexpresser and WT under salt stress vis‐à‐vis control (0 h) (k). Blue and red bars represent control (unstressed) and stressed conditions, respectively. Data are presented as means with standard error of means (*n* = 3). Bars topped with different letters represent values that are significantly different (*P* < 0.05) at 0 day or 7 day after stress.

*SaADF2*‐OE showed enhanced salt tolerance as revealed by less chlorophyll bleaching of the leaf tissues in the cut‐leaf float assay (Figure 7a) as well as seedlings in hydroponics (150 mm NaCl) (Figure 7b). As under drought stress, *SaADF2*‐OE maintained superior physiological traits over *OsADF2*‐OE and WT under salinity (Figure 7c–j). The *SaADF2*‐OE also displayed an unabated photosystem II functioning as reflected by higher Fv/Fm compared to WT (Figure 7f). *SaADF2* transcript accumulation was maintained in the leaf and root tissue of *SaADF2*‐OE under salt stress at all time points except a slight reduction at 24 h after stress (Figure 7k).

### *SaADF2* expression differed from *OsADF2* in actin filament organization under drought stress

**Figure 8:**
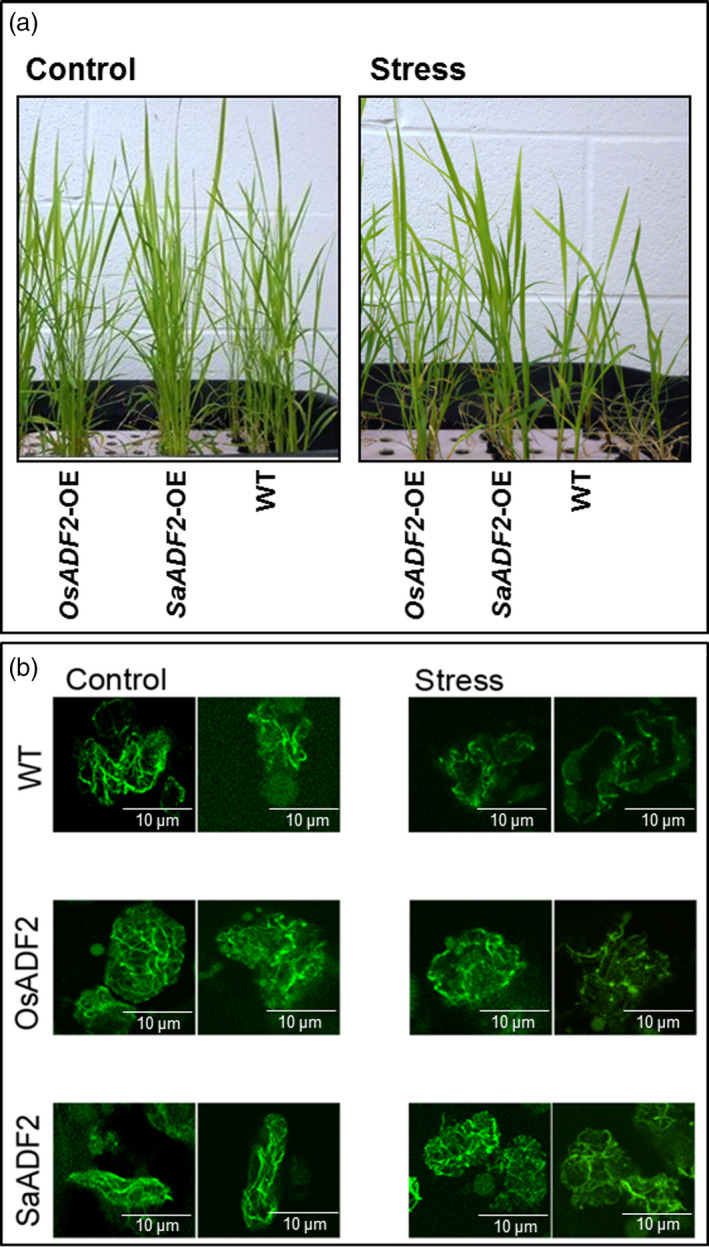
Visualization of actin filament in green plant protoplasts (b) isolated from control and stressed (mannitol, −0.3 Mpa) (a) overexpressers of *SaADF2* and *OsADF2* and WT.

Leaf mesophyll protoplasts from 10‐day‐old mannitol‐treated *ADF2*‐OE (Figure 8a) showed thick and long actin filaments (AFs) arranged longitudinally along the length of the cortical cells of the untreated control seedling (Figure 8b). However, AF organization in WT cells was significantly affected with no finer AFs, and the length of the thicker filaments was greatly reduced under osmotic stress. The small AFs, instead of adhering to organelles or being dispersed in cytosol, were shifted to the periphery closer to the plasma membrane. In *OsADF2*‐OE cells, although the mesh of cytosolic AFs was not completely lost, the integrity of fine filament structures was lost (Figure 8b). On the other hand, the number and length of filaments were considerably higher and fine filaments remained conserved in *SaADF2*‐OE cells under both control and stress. The basketlike mesh in the cytosol also remained preserved with no apparent shift of small AFs towards plasma membrane (Figure 8b).

### *SaADF2*‐OE and *OsADF2*‐OE had differential global gene expression pattern

RNA‐Seq analysis showed that unstressed control and drought stress‐induced (3 and 7 DAS) leaf transcriptome of *SaADF2*‐OE and *OsADF2*‐OE vis‐à‐vis WT resulted in 1871 significantly (log2FC ≥2 for up‐regulated and ≤−2 for down‐regulated; *P* < 0.05) differentially expressed genes (DEGs). Under drought stress, 255 genes (65 at 3 DAS and 190 at 7 DAS) were up‐regulated in *SaADF2*‐OE over *OsADF2*‐OE, whereas only 30 genes were up‐regulated under control. Altogether, 5566 genes were significantly down‐regulated (Data S1).

DEGs in *OsADF2*‐OE were significantly enriched in anion transport, photosynthesis and chlorophyll metabolism when compared to WT (Data S2). Photosynthesis was the most significantly enriched biological process, and genes predominantly localized in plastid and photosynthetic membranes, specifically in PSII, represented the cellular component. Nucleotide binding was the most enriched molecular pathway. Photosynthesis was also the most enriched biological process in *SaADF2*‐OE, but genes involved in light harvesting process, generation of precursor metabolites and energy coins were significantly enriched compared to *OsADF2*‐OE. The molecular components were concentrated in nucleotide/nucleoside binding (specifically adenosyl), phosphorylation (phosphatase and kinase) and oxidoreductase. Cellular components were mostly membrane localized.

*SaADF2*‐OE and *OsADF2*‐OE differed significantly (*P* < 0.05) for genes down‐regulated in response to abiotic stimulus (GO:000385) and response to water stress (GO:0009415). Genes in membrane integral components were significantly down‐regulated in *SaADF2*‐OE. A significantly enriched term between *SaADF2*‐OE and *OsADF2*‐OE was cation transport (GO:0006812) and others related to stress response (Data S2).

KEGG analysis also showed high enrichment of photosynthesis and general metabolism‐related pathways in *SaADF2*‐OE and *OsADF2*‐OE (Data S3). Ribosomal proteins biosynthesis was increased as a common abiotic stress response to accommodate the translation of stress‐responsive proteins. Proline and arginine metabolism path:osa00330 was also up‐regulated as a general stress response in WT/*SaADF2*‐OE at 3 DAS. Genes involved in carbohydrate biosynthetic pathways and inositol phosphate metabolism were overrepresented.

### *ADF2*‐related transcripts showed differential expression in transgenic lines

Expression analysis of DEGs, such as Ca^2+^‐dependent kinases (*CDPK/CAM Kinases*), *Rho‐GTPases*, phosphoinositide (PI) signalling‐regulated transcripts (*PI45K4*,* I145PP* and *PLD*) and protein phosphatases (Figure S4; Appendix S1), which were enriched in drought‐induced transcriptome and likely interact with *ADF2*, showed down‐regulation of most *CDPK/CAM Kinases* in *SaADF2*‐OE but fivefold to sixfold up‐regulation in WT and *OsADF2*‐OE under drought. *CAMK* isoform *AK1* (Os02g56310) was up‐regulated in *SaADF2*‐OE and *OsADF2*‐OE, but expression was lower than WT. WT and *OsADF2*‐OE accumulated eightfold and 3.5‐fold, respectively, *CAMK28* at 7 DAS, while it was down‐regulated in *SaADF2*‐OE at 7 DAS, although with 3.5‐fold accumulation over WT at 3 DAS. *Rho‐GTPases*, known to positively regulate *CDPK* activation, were down‐regulated in *SaADF2*‐OE. Phosphatases also showed down‐regulation in *SaADF2*‐OE under drought. Of the PI signalling‐regulated transcripts, *PLD* showed the same trend as *CaMK AK1*. *ADF* overexpression down‐regulated the expression of *PI4,5‐K4* and *I‐1,4,5‐PP* with no significant difference among the genotypes.

RT‐PCR of 12 putative interacting partners of *OsADF2* under drought stress in six independent *SaADF2*‐OE lines showed temporal variation in their expression profile. *SaADF2* transcript accumulation in *SaODF2*‐OE showed increase under drought stress, especially 7 DAS (Figure S5; Appendix S1). Adenyl cyclase‐associated protein (*ACP*), the known ADF‐interacting partner, demonstrated drought‐induced up‐regulation up to 1.4‐fold (D1) in *SaADF2*‐OE relative to WT.

### SaADF2 and OsADF2 interacted differently with candidate proteins

**Figure 9:**
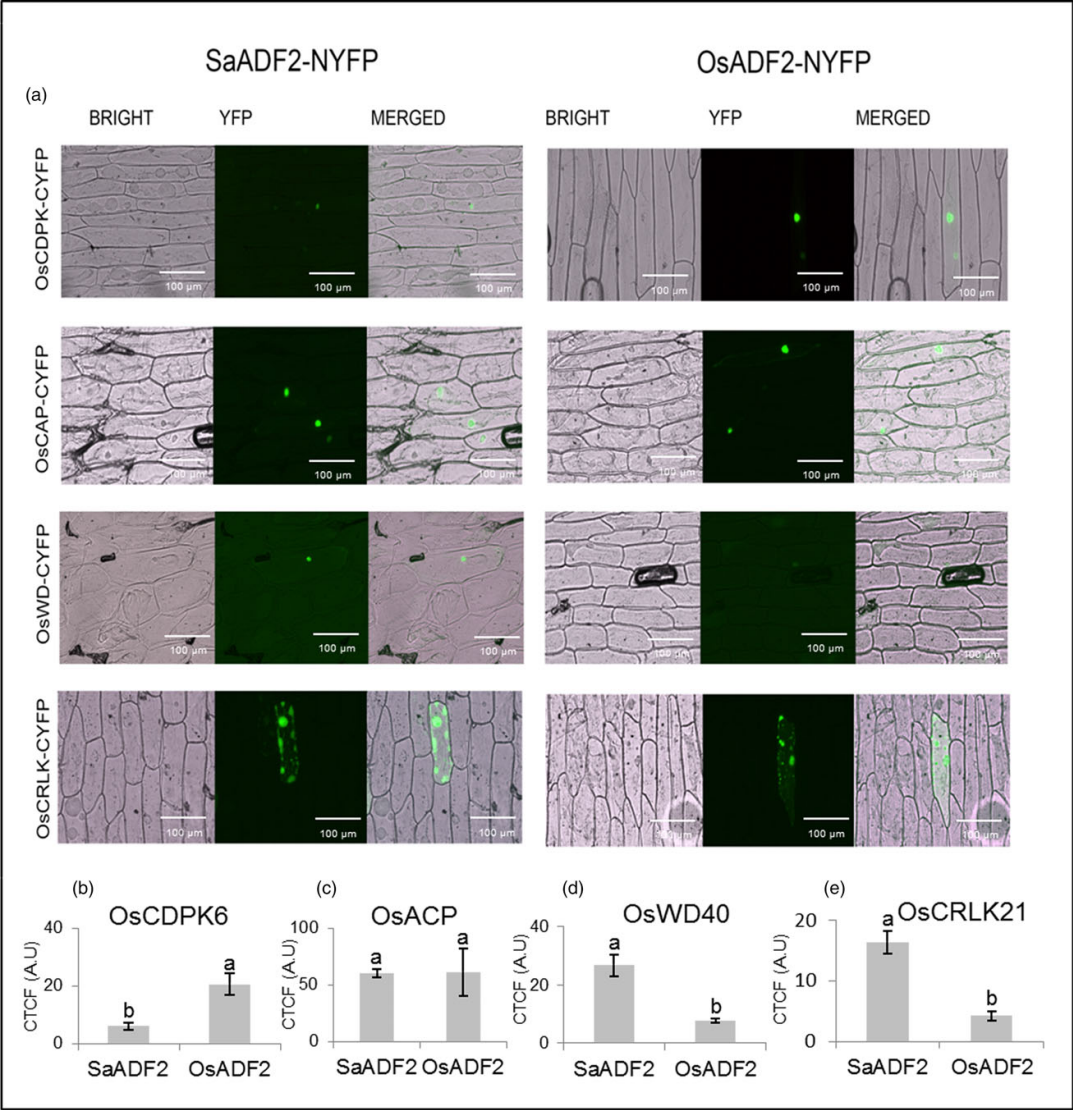
Cellular interaction pattern of SaADF2 (left panel) and OsADF2 (right panel) with four predicted interaction partners, OsCDPK6, OsCAP, OsWD40 and OsCRLK21 in onion epidermal cells using a split‐YFP system. BRIGHT—Bright‐field images, YFP—fluorescence images and MERGED—overlapped images. Bars indicate mean corrected total cell fluorescence (CTCF) ± standard deviation (*n* = 15 cells; five cells each of three independent experiments). Bars topped with different letters represent values that are significantly different (*P* < 0.05) at 0d or 7d after stress.

Bimolecular fluorescence complementation (BiFC) assay validated four ADF2‐interacting proteins, namely calcium‐dependent protein kinase (OsCDPK6, Os02g58520.1), cysteine‐rich receptor‐like protein kinase 21 (OsCRLK21, Os11g11780.1), adenylyl cyclase‐associated protein (OsACP, Os03g51250.1) and WD‐40 (G‐beta) repeat domain‐containing protein (OsWD40, Os01g03510.1) (Figure 9a). OsCDPK6 exhibited nuclear interaction with both SaADF2 and OsADF2, but substantially less fluorescence intensity for SaADF2 (Figure 9a,b). Fluorescence restitution showed that both SaADF2 and OsADF2 interacted with OsACP inside the nucleus, with no significant difference in fluorescence between the ADF2s (Figure 9a,c). SaADF2 showed nucleus‐localized interaction with OsWD40 stronger than OsADF2, which demonstrated faint fluorescence signal (Figure 9a,d). OsCRLK21 showed comparable interaction affinity with both ADF2s in cytosol, predominantly adjacent to cell membrane (Figure 9a,e). As expected, cells bombarded with only N‐terminal fragment of split‐YFP bimolecular constructs with SaADF2 or OsADF2 (negative control) did not show any fluorescence signal while optimal transactivation signal was observed in cells bombarded with constructs carrying both domains of *bHLH* transcription factor (positive control) (Figure S8; Appendix S1).

## Discussion

The present study reports biochemical and functional characterization of an actin‐depolymerizing factor (*SaADF2*) from a halophyte, *Spartina alterniflora* and its rice homolog (*OsADF2*), and that *SaADF2* overexpression imparts higher drought (and salt) tolerance in rice (as well as *Arabidopsis thaliana*; Figure S6, Appendix S1) in comparison with *OsADF2*. *ADFs* are present in multiple isoforms in higher plants (Maciver and Hussey, 2002). Of the 11 isoforms reported in rice, *OsADF2* is expressed in both vegetative and reproductive tissues without significant change in its expression under abiotic stresses (Huang *et al*., 2012). In *Physcomitrella patens*, cell viability is compromised in knockdown mutants of a single intronless *ADF* isoform (Augustine *et al*., 2008), suggesting that ADF functionality is essential for plant cells. ADF is reportedly essential for cytoskeleton rearrangement in response to extra‐ and/or intercellular stress (Ali and Komatsu, 2006; Augustine *et al*., 2008).

Superior in vitro activity of SaADF2 could be due to its longer and more exposed F‐loop than OsADF2 (Figure 1c‐e), because binding ability of ADF to F‐actin and subsequent filament severing or disassembly is attributed to the charged residues at the exposed tip of its F‐loop (Figure 1d–e) in coordination with C‐terminal α‐helix and tail (Lappalainen *et al*., 1997; Ono *et al*., 2000; Pope *et al*., 2000). F‐actin‐binding motif of ADF is highly divergent, and only a single exposed charged residue may be sufficient to effect binding (Wong *et al*., 2011), but the degree of binding may differ depending on other structural factors. SaADF2 and OsADF2, with subtle tertiary structure difference (Figure 1d), have three amino acid differences in the mostly conserved G‐actin‐binding motifs, which is comprised of N‐termini, the long α3‐helix, and the turn connecting β6 and α4/5 (Wong *et al*., 2011). This could result in their differential actin‐binding affinity (Figure 2d). Plant cells normally exhibit a slightly higher alkaline pH, and most ADFs are more active at pH ~8.0 with a few exceptions (Gungabissoon *et al*., 1998). SaADF2's ability to depolymerize actin at a broader pH range (pH 6.0–8.0) and more efficiently compared to OsADF2 along with its high actin affinity (Figure 2d–f) could prove useful to keep the plant growth and development unabated under drought (or salt) stress that frequently changes the cellular ionic concentration.

Serine‐6 in OsADF2 and its homologs from other drought and/salt tolerant/sensitive rice varieties and a halophyte wild rice (Figure S7, Appendix S1), which is substituted by threonine in SaADF2, participates in an inhibitory regulatory phosphorylation by CDPK family in plants and protists (Allwood *et al*., 2001; Smertenko *et al*., 1998). Various isoforms of CDPKs inactivate ADF by phosphorylating and inhibiting its actin binding, and consequently interfere in actin dynamics. Mutations in serine‐6 lead to the loss and/or alteration of its binding constant with CDPK that could compromise growth and development as revealed by abnormal polar tip growth of phosphomimetic and unphosphorylatable mutant protonema (Augustine *et al*., 2008). ADF interacts with CDPK in different organisms (Allwood *et al*., 2001). Less fluorescence intensity of SaADF2‐OsCDPK6 interaction indicated a physiologically more active SaADF2 protein due to a partial lift in the negative regulation of OsCDPK6 (Figure 9a,b). Lower in vitro phosphorylation efficiency of SaADF2 and OsADF2 serine‐6 mutant (OsADF2/6α) than OsADF2 by OsCDPK6 in the presence of Ca^2+^ further confirms such observation (Figure 2j–k). Thus, down‐regulation of *OsCAMKs* (Figure S4) may be functionally relevant for sustained actin dynamics in *SaADF2*‐OE.

OsWD40 is the WD‐40 (or G‐beta) repeat domain‐containing 66‐kDa stress‐regulated protein. WD domains, when present in tandem, form a propeller‐shaped scaffold useful for multiprotein interaction. WD40 has important roles in histone recognition, chromatin function, RNA processing and transcriptional regulation (Suganuma *et al*., 2008). WD repeat domain‐containing proteins, such as AIP1, disassemble the actin filaments decorated with ADF and shorten the ADF‐severed actin filaments, thus maintaining a high concentration of cellular actin monomers (Nomura *et al*., 2016). The adenylyl cyclase‐associated protein (OsACP) is highly implicated in positive regulation of actin turnover process (Ono, 2013) as an actin‐sequestering protein. ACP interacts with ADF (Zhang *et al*., 2013) for G‐actin binding, and promotes nucleotide exchange and severing of ADF‐bound actin filaments (Ono, 2013). Both interactions suggested a more active SaADF2 compared to OsADF2 in BiFC assay (Figure 9).

Amino acid substitutions could alter binding of co‐regulatory proteins, thereby changing the turnover of actin‐ADF complex in vivo. ADF may also compromise its depolymerizing activity by binding to phosphoinositide (PIP2) and inhibiting phospholipase C activity (Gungabissoon *et al*., 1998; Smertenko *et al*., 1998), thus removing itself from the cytoplasm. PIP/PIP2 binding ideally localizes plant ADF near the plasma membrane where it may participate in stress signalling (Liu *et al*., 2013; Ouellet *et al*., 2001).

Although *OsADF2* was not significantly induced under stress (Huang *et al*., 2012), *OsADF2*‐OE in the present study showed higher drought tolerance than WT. Superiority of *SaADF2*‐OE over *OsADF2*‐OE for drought (and salt) stress tolerance phenotype endorses that the difference in *in vitro* activities between highly identical SaADF2 and OsADF2 could relate to their differential response *in vivo*. Studies showing enhanced salt and/or drought tolerance of transgenics overexpressing *Spartina alterniflora* genes with subtle sequence differences from rice provide further credence (Baisakh *et al*., 2012; Joshi *et al*., 2013, 2014). Environmental perturbations restrict photosystem II (PSII) activity by inhibiting its repair, which leads to photo‐inhibition (Jin *et al*., 2011). Better vegetative and prereproductive growth of *SaADF2*‐OE under drought could be attributed to their improved photosynthetic efficiency due to less sensitivity to photo‐inhibition shown by higher Fv/Fm than *OsADF2‐*OE and WT. Quantum yield of PSII activity is directly related to chlorophyll ‘a’ (Checker *et al*., 2012). Higher chlorophyll content observed in *SaADF2*‐OE suggested more efficient internal carbon adjustment in comparison with WT and *OsADF2*‐OE under stress. Stress‐induced excessive chloroplastidic reactive oxygen species (ROS), generated as a result of imbalance between electron transport and CO_2_ fixation, reduce photosynthetic yield by dissociating or bleaching of pigment centres. Genes coding for ROS‐scavenging enzymes were up‐regulated in *SaADF2*‐OE and *OsADF2‐OE* when compared to WT, including mitochondrial superoxide dismutase, glutaredoxin, glutathione S‐transferase, peroxiredoxin, aldehyde dehydrogenase and ascorbate peroxidase (Data S1). Hence, low accumulation of O^2−^ and H_2_O_2_ in *SaADF2*‐OE may have protected the plants from membrane and PS‐II damage and photo‐inhibition under drought (Figure 5d,e). Further, maintenance of chloroplast integrity with intact grana and organized thylakoid in *SaADF2*‐OE with higher Fv/Fm under drought possibly contributed to their higher grain and biomass yield than *OSADF*‐OE and WT.

Higher RWC of *SaADF2*‐OE than *OsADF2*‐OE and WT under drought indicated greater tissue tolerance of *SaADF2*‐OE likely through superior osmotic adjustment. Transcriptome analysis (Data S1) showed up‐regulation of trehalose synthase, proline oxidase (LOC_Os10 g40360; proline dehydrogenase) and inositol synthase in *SaADF2*‐OE as compared to WT, which may be related to the protected osmotic status and conservation of relative water content of the transgenics. Additionally, group 1 and 3 LEA (late embryogenesis abundant) proteins and dehydrins that are known to impart enhanced desiccation tolerance were up‐regulated in *SaADF2‐OE* (as well as in *OsADF2*‐OE) compared to WT. Plants lose control over stomatal conductance to maintain the balance of water and gas exchange under drought and switch to flight mechanism by closing stomata to reduce water loss (Sikuku *et al*., 2010). The positive correlation between osmotic stress regulation of actin organization and K^+^ channel activity in guard cells (Luan, 2002) could explain higher stomatal conductance of *SaADF2*‐OE plants under drought stress leading to more efficient maintenance of CO_2_ exchange capacity and cellular osmotic potential than *OsADF2*‐OE and WT plants. The results indicated that drought did not affect stomatal conductance much in *SaADF2‐OE* plants, which is possibly because of their superior osmotic adjustment more by osmolyte/osmoprotectant accumulation and less by stomatal closure.

ADF increases AFs turnover through the combination of depolymerization and severing. The average length of an AF is a function of the ADF and actin monomer concentration, phosphorylation status of the subunits, availability of other ABPs (CAP or AIP) and the average time the subunit resides inside the AF. In a resting cell, fluctuation of AF length depends on the filament severing and is ~20% of average filament length (Roland *et al*., 2008). The integrity of the structural components including cytoskeleton with protected actin fibres and preserved actin mesh of the *SaADF2‐*OE could be due to maintenance of high water potential of the plants under water deficit.

Induction of *ADF* expression by salt and cold stress besides drought because of the increased rate of actin turnover suggested their role in osmoregulation (Ali and Komatsu, 2006; Baisakh *et al*., 2008; Ouellet *et al*., 2001; Salekdeh *et al*., 2002; Yang *et al*., 2003). Relative superiority of *SaADF2*‐OE over *OsADF2*‐OE and WT with better root and shoot growth under salinity is the manifestation of their better physiological responses by high RWC, membrane stability index and high Fv/Fm (Baisakh *et al*., 2012). Rapid repolymerization of AFs in cortex and nuclear envelope was recorded in cold‐treated tobacco cells (Pokorna *et al*., 2004). The significant differential expression of downstream stress‐related genes in *SaADF2*‐OE plants provides a strong indication of its role as a transactivator, in addition to modulating cytoskeleton architecture via reorganization of actin dynamics with interacting protein partners, to provide drought tolerance phenotype. Detail biochemical and functional investigation of different ADF isoforms will lead to identification of undefined molecular pathways related to cytoskeleton modulation and their precise role in abiotic stress responses (Nan *et al*., 2017).

Our data showed that ADF overexpression did not compromise with the agronomic yield of *ADF2‐OE* lines (Figure 6a‐f). On the other hand, ADF overexpression had a positive impact on important agricultural traits of transgenics at both vegetative and reproductive stages under control condition. This could be attributed to the enhanced actin dynamics in transgenics that promote cell division and expansion, and polar growth. Also, high enrichment of genes involved in photosynthesis and general metabolism‐related pathways in *ADF2*‐OE (Data S3) might have contributed to the vigour of the transgenics. Identification of the complex, differential interactome regulating stress‐modulated cytoskeleton driven by *ADF* isoforms will lead us to key genetic conduits that could be potential targets for genome engineering to improve abiotic stress resistance in crops.

## Experimental procedures

### Sequence analysis and subcellular localization of *SaADF2*

An actin‐depolymerizing factor (SaADF2) from the salt‐induced transcriptome of *Spartina alterniflora* (Baisakh and Mangu, 2016; Bedre *et al*., 2016) was queried against the NCBI and UniProtKB nonredundant database. *SaADF2* and orthologs from rice and *Arabidopsis* were aligned, and phylogenetic tree was constructed using CLC workbench v7.0. Homology‐based threading was performed in I‐TASSER stand‐alone server (Yang *et al*., 2015) or LOMETS (Wu and Zhang, 2007) and predicted three‐dimensional structures were analysed and aligned using UCSF Chimera (Pettersen *et al*., 2004).

*SaADF2* was cloned in frame with green fluorescent protein gene (*gfp*) under 35S promoter at *Nco* I and *Spe* I sites of pCAMBIA1302, and the resulting P35S::*SaADF2*:*gfp* fusion construct was bombarded into onion epidermal cells to visualize subcellular localization as described by Baisakh *et al*. (2012).

### Expression and purification of recombinant ADF2 proteins

Full‐length cDNA of *SaADF2*,* OsADF2* (LOC_Os03 g56790) and OsCDPK6 (LOC_Os02 g58520.1) was cloned in pET200 carrying an N‐terminal His‐tag to generate pET200‐SaADF2/OsADF2 using the standard Gateway technology (Invitrogen, Carlsbad, CA). A point mutant 6α was generated at the serine‐6, the major phosphorylation site in OsADF2 by substituting serine with threonine to mimic SaADF2 using In‐Fusion cloning kit (Clontech, Paolo Alto, CA) in pET200‐SaADF2/OsADF2 according to the manufacturer's instructions. The recombinant proteins were expressed in *Escherichia coli* BL21 (DE3) and purified following standard protocol (Method S1). The affinity tag was removed from the recombinant proteins with thrombin restriction 3 (EMD Millipore, Chicago, IL) for all downstream biochemical analyses, except phosphorylation.

### Actin polymerization and co‐sedimentation assay

Human platelet G‐actin (Cytoskeleton Inc., Denver, CO) was polymerized at RT following the manufacturer's protocol. The F‐actin/actin bundles were separated from the G‐actin by centrifugation (40 000 ***g***) at 4 °C for 3 h. The pellet was reconstituted in actin‐binding buffer (ABB; 10 mm Tris, 1 mm ATP, 0.2 mm DTT, 1 mm EGTA, 0.1 mm CaCl_2_ and 2 mm MgCl_2_), and used immediately for binding assays. Low‐speed co‐sedimentation of SaADF2 and OsADF2 with actin was performed as described by Allwood *et al*. (2001) (Method S1).

### F‐actin depolymerization assay and visualization of actin disassembly and severing

Four micromolar rabbit muscle 30% pyrene‐labelled actin (Cytoskeleton, Inc.) was polymerized as described by Singh *et al*. (2011). F‐actin depolymerization was induced with 0.8 μm SaADF2/OsADF2 either by using prepolymerized actin or by adding proteins to an actively polymerizing G‐actin following Carlier *et al*. (1997) (Method S1). Actin filament disassembly and severing by ADF proteins was observed by total internal reflection fluorescence (TIRF) microscopy, as described by Shekhar and Carlier (2017), with modifications (Method S1).

### In vitro phosphorylation

In vitro phosphorylation was performed following in the presence of 4 μm CDPK, 16 μm ADF and 4 μm ATP following Allwood *et al*. (2001). All proteins were dephosphorylated with calf intestinal phosphatase (CIP, New England Biolabs, Ipswich, MA) prior to phosphorylation. Following phosphorylation, His‐tagged ADF was immunoprecipitated with anti‐His antibody and protein A/G sepharose (Pierce, Waltham, MA), eluted in low pH, and dialysed (Methods S1). The protein fractions were immunoblotted with antiphosphoserine antibody (Abcam, Cambridge, MA). The membrane was CIP‐treated prior to blocking with rabbit serum. Membrane was developed using ECL chemiluminescence kit (Pierce) following the manufacturer's instructions.

### Construction of binary vector and development of rice transgenics

First‐strand cDNA was synthesized from total RNA isolated from *S. alterniflora* and rice as described in Baisakh *et al*. (2012). The complete coding sequence of *SaADF2* and *OsADF2* was amplified from the respective first‐strand cDNA using forward and reverse primers containing *Bgl* II and *Bst* EII restriction endonuclease recognition sites, respectively (Table S1). Construction of p35S:*SaADF2/OsADF2* in pCAMBIA1305.1 backbone and its subsequent mobilization into *Agrobacterium tumefaciens* LBA4404 was performed following Baisakh *et al*. (2012).

*Agrobacterium tumefaciens*‐mediated transformation of rice cultivar ‘Nipponbare’ was performed following Rao *et al*. (2009). Primary transgenics, confirmed using *SaADF2/OsADF2* gene‐specific primers (Method S1, Table S1), were seed‐advanced to T_2_ generation for achieving homozygosity.

### Drought and salinity tolerance assay

Drought stress was imposed on 50‐d‐old homozygous progenies of *SaADF2*‐OE#23, 38, and 41, *OsADF2*‐OE#2, 5, 20, and WT as described earlier (Joshi *et al*., 2014; Method S1). Three‐week‐old seedlings of homozygous progenies of *SaADF2*‐OE, *OsADF2*‐OE and WT grown in hydroponics with Yoshida's nutrient solution (Yoshida *et al*., 1976) were subjected to salt stress (150 mm NaCl) for a week as described earlier (Baisakh *et al*., 2012).

### Phenotypic, physiological, biochemical and microscopic analyses

Control and stressed plants were observed for common stress‐induced phenotypes, and physiological traits were measured following the procedures described earlier (Baisakh *et al*., 2012; Joshi *et al*., 2014). O^2−^ and H_2_O_2_ were visualized in situ following Jabs *et al*. (1996) and Thordal‐Christensen *et al*. (1997), respectively. Transmission electron microscopy (Marques *et al*., 2018) and scanning electron microscopy (Baisakh *et al*., 2012) were conducted to examine chloroplast ultrastructure and stomata, respectively (Method S1). All quantitative data were analysed statistically for variance (ANOVA), and treatment means were compared by Tukey's HSD in XLSTAT add‐in of Microsoft Excel.

### (Semi)‐quantitative reverse transcription PCR

RT‐PCR was conducted using first‐strand cDNA from leaf and root tissues of control and stressed *SaADF2*‐OE and WT plants as described earlier (Baisakh *et al*., 2012; Method S1) to study the expression of *SaADF2* and genes, which were overrepresented in the comparative transcriptome analysis (*SaADF2*‐OE vs *OsADF2*‐OE and WT) and selected from the network analysis using STRING (www.stringdb.org) and RiceNet v2 (Lee *et al*., 2011) after excluding the hypothetical and ribosomal proteins, using gene‐specific primers (Table S1).

### Leaf protoplast isolation and staining of actin filaments

Green protoplasts were isolated from 10‐d‐old control and mannitol‐stressed (equivalent with water stress to ѱ_os_ = −0.3 MPa) seedlings of WT, *SaADF2*‐OE, and *OsADF2*‐OE following Zhang *et al*. (2011). Twenty microlitre of intact protoplast suspensions was permeabilized on poly‐L‐lysine‐coated slides in a humid chamber with 3% Triton X‐100 in PBS (pH 7.4). Actin staining with 5 IU of Alexa Fluor 488‐Phalloidin (Cytoskeleton Inc) was performed following Zhao *et al*. (2011), and optical sections in Z‐stacks at 1 μm interval were taken by LSM700 (Zeiss; 40x/1.3 objective; Method S1).

### Bimolecular fluorescence (BiFC) complementation

Bimolecular fluorescence (BiFC) complementation was performed using the method described by Pattanaik *et al*. (2011). Split‐YFP vectors, pA7‐*SaADF2*/NYFP and pA7‐*OsADF2*/NYFP containing SaADF2 and OsADF2 fused with N‐terminal end of YFP, and pA7‐interacting protein(s) fused with C‐terminal end of YFP, were constructed (Method S1) and delivered by a particle gun into onion epidermal cells at 1100 psi following Baisakh *et al*. (2012). GFP fluorescence was observed after 22 h under blue light with an Olympus SZH10 GFP‐stereomicroscope equipped with a Nikon DXM1200C camera and ACT‐1 software.

### Genomewide transcriptome analysis of *SaADF2*‐OE vis‐à‐vis *OsADF2*‐OE and WT

RNA‐Seq libraries were prepared from unstressed (control) and 3 and 7 DAS WT, *SaADF2*‐OE and *OsADF2*‐OE in three biological replicates and sequenced in an Illumina HiSeq 2000 platform with 150‐cycle paired‐end as described in Bedre *et al*. (2015). A total of 706 904 570 sequence reads (70.69 Gbp) were generated that corresponded to 623.83X coverage of the transcriptome (raw reads deposited in NCBI SRA database, Acc. No. PRJNA393177). Downstream sequence manipulations, such as filtering, mapping, assembly, differential gene expression, GO and KEGG analyses, were performed following Bedre *et al*. (2016).

## Conflict of interest

The authors have no conflict of interest to declare.

## Supporting information


**Figure S1** Nuclear localization of SaADF2.
**Figure S2** SDS‐PAGE analysis of N‐terminal 6X‐His‐tagged OsADF2 (a), SaADF2 (b) and OsADF2/6α after ammonium sulphate precipitation and Ni‐NTA resin purification. CL; Cell Lysate, FT; Column Flow Through, W1/2; Wash 1/2, F1; Fraction 1, 200 mm Imidazole eluate containing purified protein; F2; Fraction 2, 250 mm Imidazole eluate containing purified protein, M; Molecular Weight Marker. Immunoblot from soluble and membrane fractions of *E. coli* cell lysate expressing OsADF2, SaADF2 or OsADF2/6α recombinant proteins with monoclonal anti‐His antibody (c). US; Uninduced Supernatant fraction, UP; Uninduced Pellet fraction, IS; Induced Supernatant fraction, IP; Induced Pellet fraction, S; Supernatant, P; Pellet. BL21 cell lysate was used as negative control.
**Figure S3** Drought tolerance of the SaADF2‐overexpressing transgenics 7 DAS (a), 11 DAS (b), and 14 DAS (c) compared to WT. Recovery of the 14d‐stressed SaADF2‐overexpressing transgenics and WT after 4 d of resuming irrigation (d), 11d‐stressed flowering plants 14 and 28 days after recovery (e, f). In the absence of stress, WT and SaADF2‐overexpressing plants have similar growth and reproduction (g). Plastid arrangement of WT and SaADF2‐overexpressing transgenic line under drought stress (h). Soil moisture content of the soil 7 DAS (i). Stomatal mean aperture (j) C=control, S=Stress.
**Figure S4** Quantitative real‐time PCR profile of functionally important genes enriched in RNA‐seq data. phosphatidylinositol‐4‐phosphate 5‐kinase, PI45K4; histidine acid phosphatase, HIP; protein phosphatase 2C, PP2C1; type I inositol‐1,4,5‐trisphosphate 5‐phosphatase, I145PP; phosphatidic acid phosphatase‐related, PAP; protein phosphatase 2C, PP2C2; protein phosphatase 2C, PP2C3; mitochondrial Rho‐GTPase 1, mRho1; rhoGAP domain‐containing protein, Rho; rho‐GTPase‐activating protein‐related, RhoL; CAMK_KIN1/SNF1/Nim1_like.8 ‐ CAMK includes calcium/calmodulin dependent protein kinases, CAMK8; CAMK_CAMK_like 7, CAMK7; calcium‐dependent protein kinase isoform AK1, AK1; CAMK_KIN1/SNF1/Nim1_like.15, CAMK15; CAMK_KIN1/SNF1/Nim1_like. 26, CAMK26; CAMK_CAMK_like.20, CAMK20; CAMK_KIN1/SNF1/Nim1_like.3, CAMKL3; CAMK_KIN1/SNF1/Nim1_like.30, CAMK30; CAMK_KIN1/SNF1/Nim1_like.28, CAMK28; PhospholipaseD, PLD. WT, wild type, Sa, SaADF2, Os, OsADF2; 0,3, an7d denote 0, 3, and 7 days after stress.
**Figure S5** Predicted filtered interactome map of SaADF2/OsADF2 constructed using RiceNet v2 (a). b. Semiquantitative expression analysis of representative interactive partners under control (D0) and 1 day (D1), 3 days (D3), and 7 days (D7) after drought stress in WT and six independent lines of SaADF2‐overexpressing transgenics. WD domain G‐beta repeat domain‐containing Protein/ At5 g58230 MSI1 (MSI, MULTICOPY SUPRESSOR OF IRA1), GTP‐binding protein (OsRAc1), mitochondrial heat shock protein ((mtHSP70‐1, mtHSP70‐2), chloroplastidic heat shock protein (cHSP70‐4), Copper/Zinc superoxide dismutase1 (C/Z‐SD1, C/Z‐SD2), Adenyl cyclase‐associated protein (ACP), T‐complex protein, putative, expressed (TCP), CS domain‐containing protein (CS).
**Figure S6** *SaADF2* overexpression conferred salt (a) and drought tolerance to *Arabidopsis* transgenics as compared with wild type (WT). Salt (100 mm NaCl) and drought stress (withholding irrigation) was imposed on 3‐week seedlings until flowering and seed setting.
**Figure S7** Alignment of ADF2 amino acid sequences from Nipponbare, Nagina 22 (N22), *Porteresia coarctata* (Por), IR29, Pokkali (Pok), Geumgbyeo (Geu), Nonabokra (NB), Cocodrie (Coco), Vandana (Van) and IR64.
**Figure S8** Negative (a) and positive (b) control for BiFC. Only‐N‐terminal fragments of split‐YFP bimolecular constructs carrying SaADF2 and OsADF2 was bombarded as a negative control. And as a positive control, bombarded transactivation domains of *bHLH* TF with the same construct was bombarded (Pattanaik *et al*., 2011).

**Table S1** Sequences of primers used in the study.

**Appendix S1** Descriptive legends to supporting figures.

**Data S1** Comparison of differentially expressed genes among WT, *SaADF2*‐OE and *OsADF2*‐OE).

**Data S2** Gene ontology of genes differentially expressed in *ADF2*‐overexpressers vis‐à‐vis WT under control and drought.

**Data S3** KEGG enrichment analysis for differentially expressed genes.

**Method S1** Supporting experimental procedures.
