# Supplementary material for: An actin‐depolymerizing factor from the halophyte smooth cordgrass, Spartina alterniflora (SaADF2), is superior to its rice homolog (OsADF2) in conferring drought and salt tolerance when constitutively overexpressed in rice
Source: Plant Biotechnol J. 2018 Jun 28;17(1):188–205. doi: 10.1111/pbi.12957 (PMC6330539; doi:10.1111/pbi.12957)
Supplement: Supplementary file 1 — Figure S1 Nuclear localization of SaADF2. Figure S2 SDS‐PAGE analysis of N‐terminal 6X‐His‐tagged OsADF2 (a), SaADF2 (b) and OsADF2/6α after ammonium sulphate precipitation and Ni‐NTA resin purification. CL; Cell Lysate, FT; Column Flow Through, W1/2; Wash 1/2, F1; Fraction 1, 200 mm Imidazole eluate containing purified protein; F2; Fraction 2, 250 mm Imidazole eluate containing purified protein, M; Molecular Weight Marker. Immunoblot from soluble and membrane fractions of E. coli cell lysate expressing OsADF2, SaADF2 or OsADF2/6α recombinant proteins with monoclonal anti‐His antibody (c). US; Uninduced Supernatant fraction, UP; Uninduced Pellet fraction, IS; Induced Supernatant fraction, IP; Induced Pellet fraction, S; Supernatant, P; Pellet. BL21 cell lysate was used as negative control. Figure S3 Drought tolerance of the SaADF2‐overexpressing transgenics 7 DAS (a), 11 DAS (b), and 14 DAS (c) compared to WT. Recovery of the 14d‐stressed SaADF2‐overexpressing transgenics and WT after 4 d of resuming irrigation (d), 11d‐stressed flowering plants 14 and 28 days after recovery (e, f). In the absence of stress, WT and SaADF2‐overexpressing plants have similar growth and reproduction (g). Plastid arrangement of WT and SaADF2‐overexpressing transgenic line under drought stress (h). Soil moisture content of the soil 7 DAS (i). Stomatal mean aperture (j) C=control, S=Stress. Figure S4 Quantitative real‐time PCR profile of functionally important genes enriched in RNA‐seq data. phosphatidylinositol‐4‐phosphate 5‐kinase, PI45K4; histidine acid phosphatase, HIP; protein phosphatase 2C, PP2C1; type I inositol‐1,4,5‐trisphosphate 5‐phosphatase, I145PP; phosphatidic acid phosphatase‐related, PAP; protein phosphatase 2C, PP2C2; protein phosphatase 2C, PP2C3; mitochondrial Rho‐GTPase 1, mRho1; rhoGAP domain‐containing protein, Rho; rho‐GTPase‐activating protein‐related, RhoL; CAMK_KIN1/SNF1/Nim1_like.8 ‐ CAMK includes calcium/calmodulin dependent protein kinases, CAMK8; CAMK_CAMK_l [file PBI-17-188-s007.pdf]

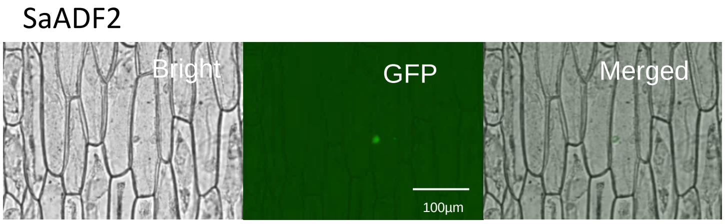

Figure S1. Nuclear localization of SaADF2

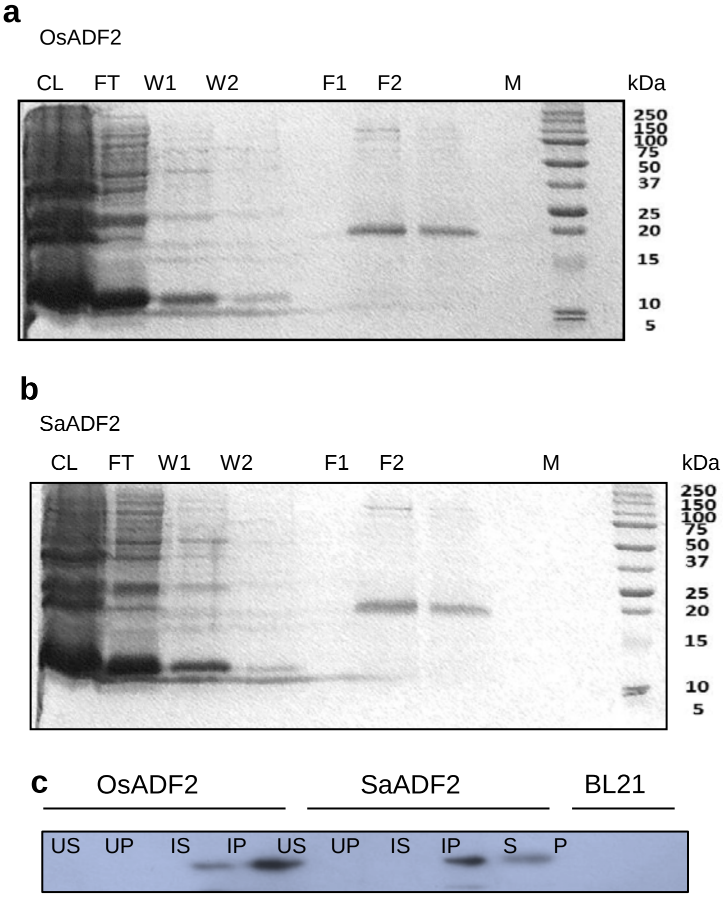

Figure S2. SDS-PAGE analysis of N-terminal 6X-his tagged OsADF2 (a), SaADF2 (b) and OsADF2/6α after ammonium sulphate precipitation and Ni-NTA resin purification. CL; Cell Lysate, FT; Column Flow Through, W1/2; Wash 1/2, F1; Fraction 1, 200mM Imidazole eluate containing purified protein; F2; Fraction 2, 250mM Imidazole eluate containing purified protein, M; Molecular Weight Marker. Immunoblot from soluble and membrane fractions of E.coli cell lysate expressing OsADF2, SaADF2 or OsADF2/6α recombinant proteins with monoclonal anti-his antibody (c). US; Uninduced Supernatant fraction, UP; Uninduced Pellet fraction, IS; Induced Supernatant fraction, IP; Induced Pellet fraction, S; Supernatant, P; Pellet. BL21 cell lysate was used as negative control.

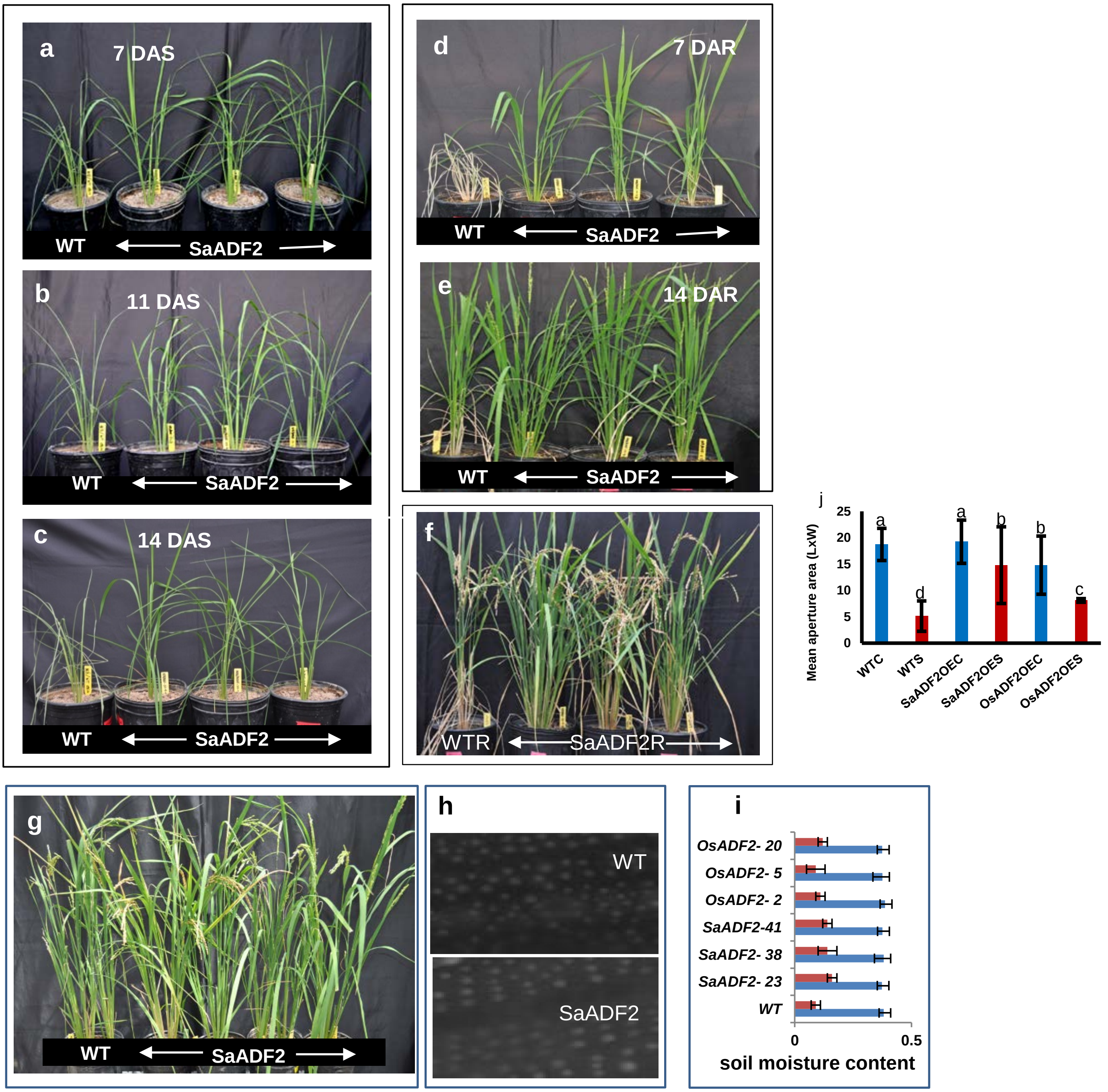

Figure S3. Drought tolerance of the SaADF2-overexpressing transgenics 7 DAS (a), 11 DAS (b), and 14 DAS (c) compared to WT. Recovery of the 14d-stressed SaADF2-overexpressing transgenics and WT after 4 d of resuming irrigation (d), 11d-stressed flowering plants 14 and 28 days after recovery (e, f). In the absence of stress, WT and SaADF2-overexpressing plants have similar growth and reproduction (g). Plastid arrangement of WT and SaADF2-overexpressing transgenic line under drought stress (h). Soil moisture content of the soil 7 DAS (i). Stomatal mean aperture (j) C=control, S=Stress.

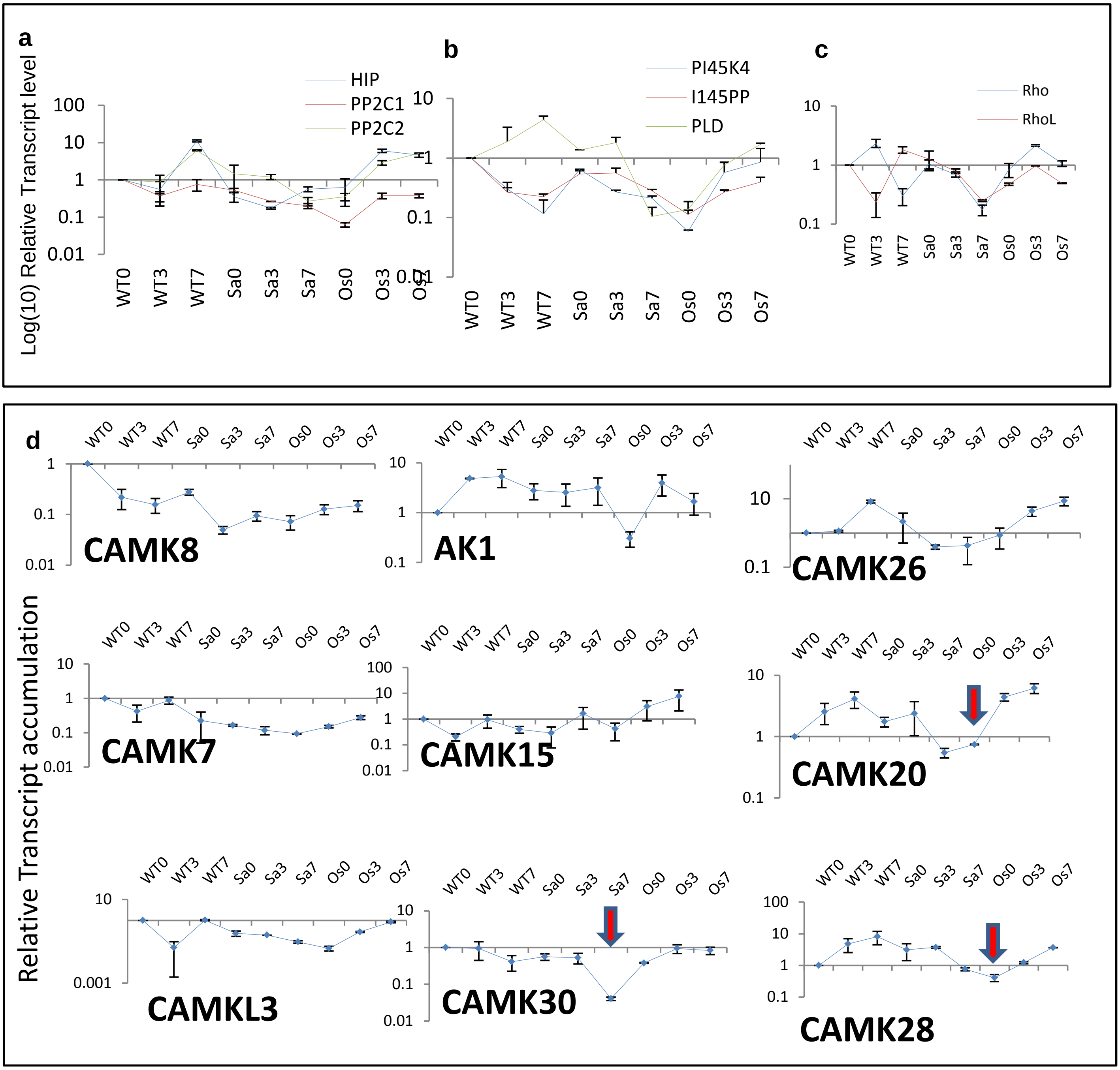

Figure S4. Quantitative real-time PCR profile of functionally important genes enriched in RNA-seq data. phosphatidylinositol-4-phosphate 5-kinase, PI45K4; histidine acid phosphatase, HIP; protein phosphatase 2C, PP2C1; type I inositol-1,4,5-trisphosphate 5-phosphatase, I145PP; phosphatidic acid phosphatase-related, PAP; protein phosphatase 2C, PP2C2; protein phosphatase 2C, PP2C3; mitochondrial Rho GTPase 1, mRho1; rhoGAP domain containing protein, Rho; rho-GTPase-activating protein-related, RhoL; CAMK_KIN1/SNF1/Nim1_like.8 - CAMK includes calcium/calmodulin dependent protein kinases, CAMK8; CAMK_CAMK_like 7, CAMK7; calcium-dependent protein kinase isoform AK1, AK1; CAMK_KIN1/SNF1/Nim1_like.15, CAMK15; CAMK_KIN1/SNF1/Nim1_like. 26, CAMK26; CAMK_CAMK_like.20, CAMK20; CAMK_KIN1/SNF1/Nim1_like.3, CAMKL3; CAMK_KIN1/SNF1/Nim1_like.30, CAMK30; CAMK_KIN1/SNF1/Nim1_like.28, CAMK28; PhospholipaseD, PLD. WT, wild type, Sa, SaADF2, Os, OsADF2; 0,3, an7d denote 0,3, and 7 days after stress.

a

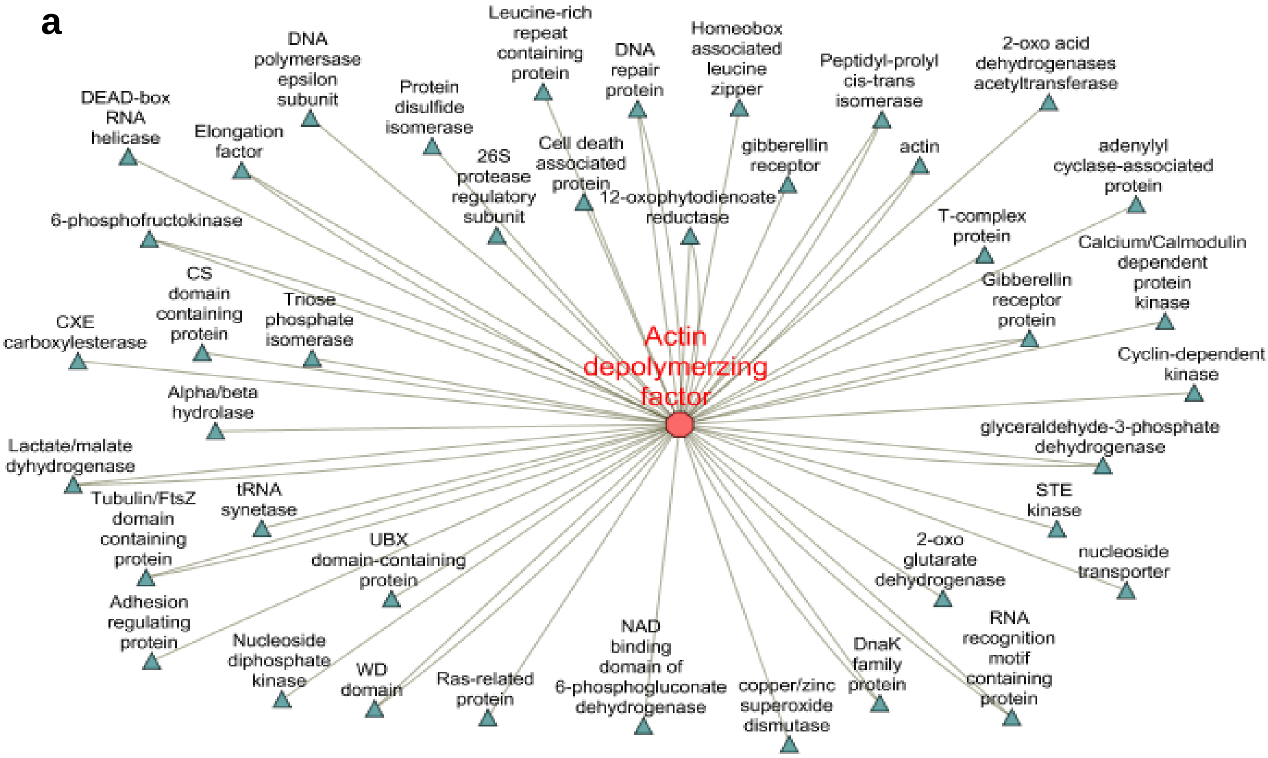

b

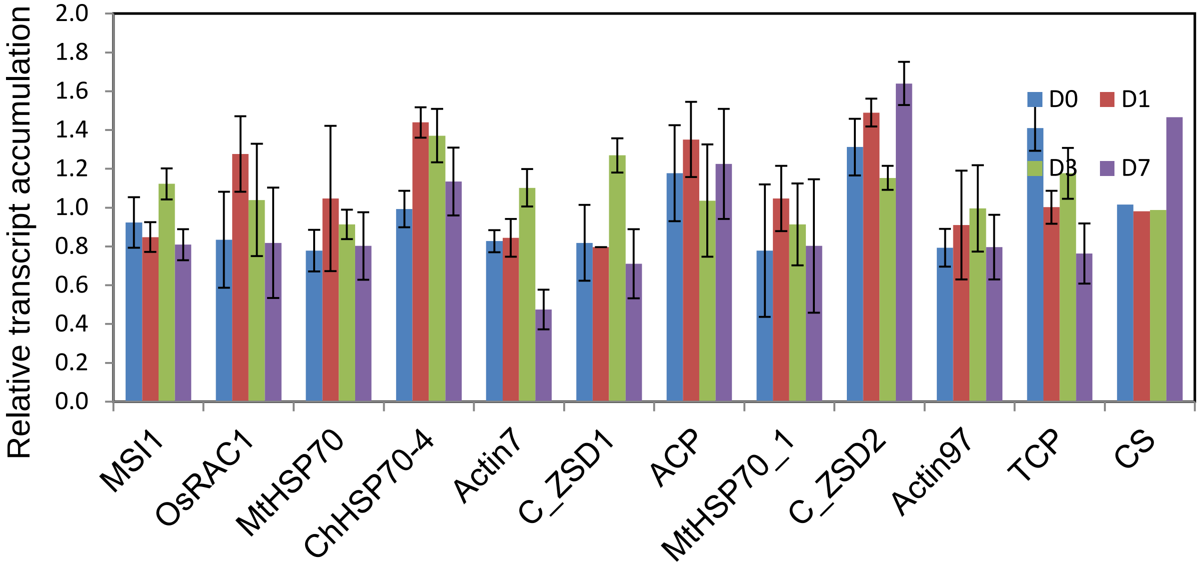

Figure S5. Predicted filtered interactome map of SaADF2/OsADF2 constructed using RiceNet v2 (a). b. Semiquantitative expression analysis of representative interactive partners under control (D0) and 1 day (D1), 3 days (D3), and 7 days (D7) after drought stress in WT and six independent lines of SaADF2-overexpressing transgenics. WD domain G-beta repeat domain containing Protein/ At5g58230 MSI1 (MSI, MULTICOPY SUPRESSOR OF IRA1), GTP-binding protein (OsRAc1), mitochondrial heat shock protein ((mtHSP70-1, mtHSP70-2), chloroplastidic heat shock protein (cHSP70-4), Copper/Zinc superoxide dismutase1 (C/Z-SD1, C/Z-SD2), Adenyl cyclase-associated protein (ACP), T-complex protein, putative, expressed (TCP), CS domain containing protein (CS).

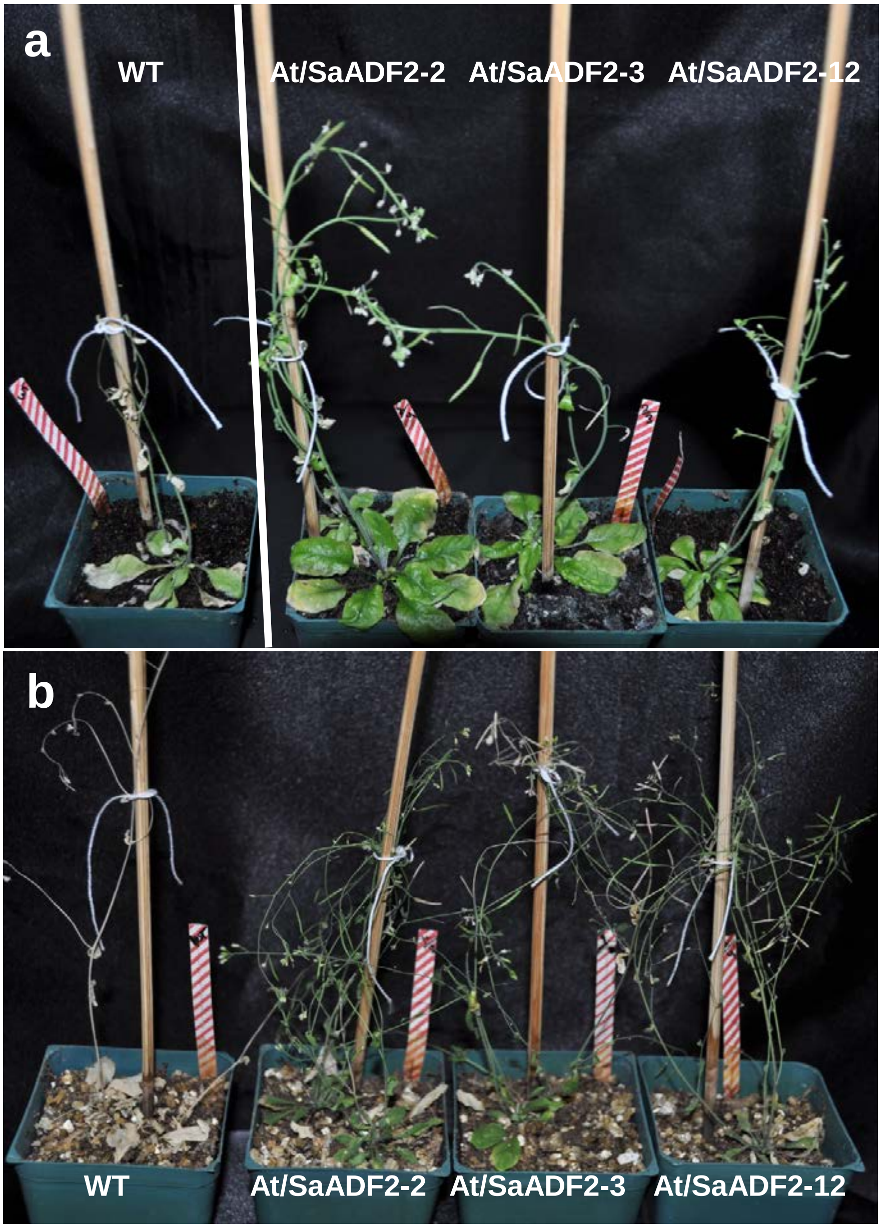

Figure S6. *SaADF2* overexpression conferred salt (a) and drought tolerance to *Arabidopsis* transgenics as compared with wild type (WT). Salt (100 mM NaCl) and drought stress (withholding irrigation) was imposed on 3-week seedlings until flowering and seed setting.

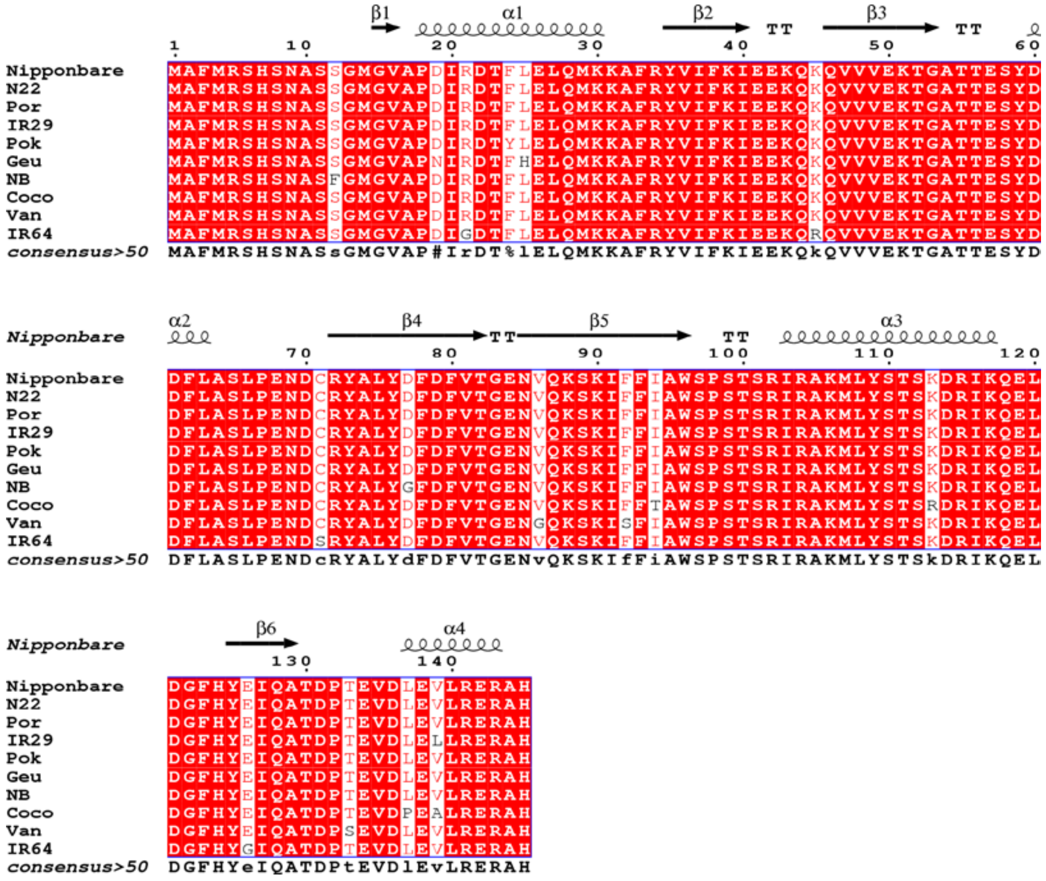

Figure S7. Alignment of ADF2 amino acid sequences from Nipponbare, Nagina 22 (N22), *Porteresia coarctata* (Por), IR29, Pokkali (Pok), Geumgbyeo (Geu), Nonabokra (NB), Cocodrie (Coco), Vandana (Van) and IR64.

a b

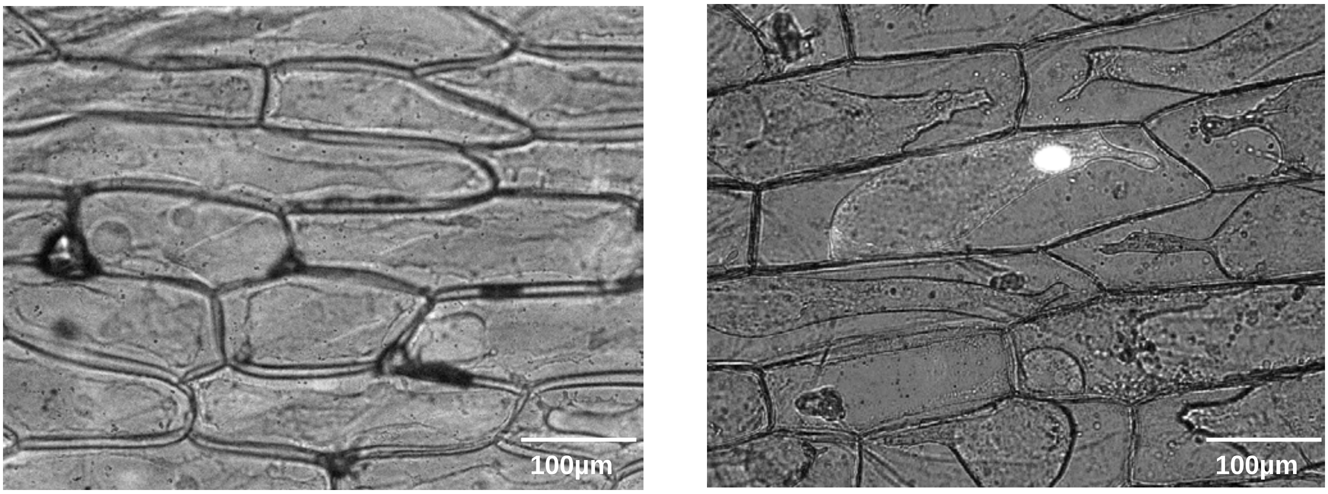

N-YFP-SaADF2 N-YFP-Myb/C-YFP-BHLH

Figure S8. Negative (a) and positive (b) control for BiFC. Only-N-terminal fragments of split-YFP bimolecular constructs carrying SaADF2 and OsADF2 was bombarded as a negative control. And as a positive control, bombarded transactivation domains of *bHLH* TF with the same construct was bombarded (Pattanaik *et al*. 2011)
