# Supplementary material for: An actin‐depolymerizing factor from the halophyte smooth cordgrass, Spartina alterniflora (SaADF2), is superior to its rice homolog (OsADF2) in conferring drought and salt tolerance when constitutively overexpressed in rice
Source: Plant Biotechnol J. 2018 Jun 28;17(1):188–205. doi: 10.1111/pbi.12957 (PMC6330539; doi:10.1111/pbi.12957)
Supplement: Supplementary file 3 — Appendix S1 Descriptive legends to supporting figures. [file PBI-17-188-s005.docx]

**Appendix S1. Descriptive Legends to Supporting Figures**

Fig. S2. SDS-PAGE analysis of N-terminal 6X-his tagged OsADF2 (a), SaADF2 (b) and OsADF2/6α after ammonium sulphate precipitation and Ni-NTA resin purification. CL; Cell Lysate, FT; Column Flow Through, W1/2; Wash 1/2, F1; Fraction 1, 200mM Imidazole eluate containing purified protein; F2; Fraction 2, 250mM Imidazole eluate containing purified protein, M; Molecular Weight Marker. Immunoblot from soluble and membrane fractions of E.coli cell lysate expressing OsADF2, SaADF2 or OsADF2/6α recombinant proteins with monoclonal anti-his antibody (c). US; Uninduced Supernatant fraction, UP; Uninduced Pellet fraction, IS; Induced Supernatant fraction, IP; Induced Pellet fraction, S; Supernatant, P; Pellet. BL21 cell lysate was used as negative control.

Fig. S3. Drought tolerance of the SaADF2-overexpressing transgenics 7 DAS (a), 11 DAS (b), and 14 DAS (c) compared to WT. Recovery of the 14d-stressed SaADF2-overexpressing transgenics and WT after 4 d of resuming irrigation (d), 11d-stressed flowering plants 14 and 28 days after recovery (e, f). In the absence of stress, WT and SaADF2-overexpressing plants have similar growth and reproduction (g). Plastid arrangement of WT and SaADF2-overexpressing transgenic line under drought stress (h). Soil moisture content of the soil 7 DAS (i).

Fig. S4. Quantitative real-time PCR profile of functionally important genes and transcripts enriched in RNA-seq data.

phosphatidylinositol-4-phosphate 5-kinase, putative, expressed PI45K4

histidine acid phosphatase, putative, expressed HIP

protein phosphatase 2C, putative, expressed PP2C1

type I inositol-1,4,5-trisphosphate 5-phosphatase, putative, expressed I145PP

phosphatidic acid phosphatase-related, putative, expressed PAP

protein phosphatase 2C, putative, expressed PP2C2

protein phosphatase 2C, putative, expressed PP2C3

mitochondrial Rho GTPase 1, putative, expressed mRho1

rhoGAP domain containing protein, expressed Rho

rho-GTPase-activating protein-related, putative, expressed RhoL

CAMK_KIN1/SNF1/Nim1_like.8 - CAMK includes calcium/calmodulin depedent protein kinases, expressed CAMK8CAMK_CAMK_like.7 - CAMK includes calcium/calmodulin depedent protein kinases, expressed CAMK7

calcium-dependent protein kinase isoform AK1, putative, expressed AK1

CAMK_KIN1/SNF1/Nim1_like.15 - CAMK includes calcium/calmodulin depedent protein kinases, expressed CAMK15

CAMK_KIN1/SNF1/Nim1_like.26 - CAMK includes calcium/calmodulin depedent protein kinases, expressed CAMK26

CAMK_CAMK_like.20 - CAMK includes calcium/calmodulin depedent protein kinases, expressed CAMK20

CAMK_KIN1/SNF1/Nim1_like.3 - CAMK includes calcium/calmodulin depedent protein kinases, expressed CAMKL3

CAMK_KIN1/SNF1/Nim1_like.30 - CAMK includes calcium/calmodulin depedent protein kinases, expressed CAMK30

CAMK_KIN1/SNF1/Nim1_like.28 - CAMK includes calcium/calmodulin depedent protein kinases, expressed CAMK28

PhospholipaseD PLD

Fig. S5. Predicted filtered interactome map of SaADF2/OsADF2 constructed using RiceNet v2 (a). b. Semiquantitative expression analysis of representative interactive partners under control (D0) and 1 day (D1), 3 days (D3), and 7 days (D7) after drought stress in WT and six independent lines of SaADF2-overexpressing transgenics. Elongation factor internal control (OsEF1a), glyceraldehyde-3-phosphate dehydrogenase (G3PD), WD domain G-beta repeat domain containing Protein/ At5g58230 MSI1 (MSI, MULTICOPY SUPRESSOR OF IRA1), GTP-binding protein (OsRAc1), mitochondrial heat shock protein ((mtHSP70-1, mtHSP70-2), chloroplastidic heat shock protein (cHSP70-4), G-beta repeat domain containing protein (66Str-pro), peptidyl-prolyl cis-trans isomerase (CYCLOPHILIN2), Copper/Zinc superoxide dismutase1 (C/Z-SD1, C/Z-SD2), Adenyl cyclase-associated protein (ACP), Lactate/malate dehydrogenase, putative (MDH1), T-complex protein, putative, expressed (TCP), CS domain containing protein (CS).

Fig. S6. *SaADF2* overexpression conferred salt (a) and drought tolerance to *Arabidopsis* transgenics as compared with wild type (WT). Salt (100 mM NaCl) and drought stress (withholding irrigation) was imposed on 3-week seedlings until flowering and seed setting.

Fig. S7. Alignment of ADF2 amino acid sequences from Nipponbare, Nagina 22 (N22), *Porteresia coarctata* (Por), IR29, Pokkali (Pok), Geumgbyeo (Geu), Nonabokra (NB), Cocodrie (Coco), Vandana (van) and IR64.
