# Supplementary material for: An actin‐depolymerizing factor from the halophyte smooth cordgrass, Spartina alterniflora (SaADF2), is superior to its rice homolog (OsADF2) in conferring drought and salt tolerance when constitutively overexpressed in rice
Source: Plant Biotechnol J. 2018 Jun 28;17(1):188–205. doi: 10.1111/pbi.12957 (PMC6330539; doi:10.1111/pbi.12957)
Supplement: Supplementary file 7 — Method S1 Supporting experimental procedures. [file PBI-17-188-s004.docx]

**Supporting Information Method S1**

**An Actin Depolymerizing Factor from the Halophyte Smooth Cordgrass, *Spartina alterniflora* (*SaADF2*) is Superior to its Rice homolog (*OsADF2*) in Conferring Drought and Salt Tolerance when Constitutively Overexpressed in Rice**

**Sengupta et al.**

**Expression and purification of recombinant ADF2 proteins**

Full length cDNA of *SaADF2* and *OsADF2* (LOC_Os03g56790) was cloned in the prokaryotic expression vector, pET200 carrying an N-terminal His-tag (Invitrogen, Carlsbad, CA) using the Standard Gateway technology. The recombinant proteins were expressed in *Escherichia coli* BL21 (DE3) in the presence of 1 mM IPTG and confirmed by immune-detection with anti-his antibody (GenWay Biotech Inc., San Diego, CA).The proteins were purified (~60% purification) with Ni-NTA resin columns (Qiagen, Valencia, CA) under a mild acidic condition (pH 5-6) with 200-250 mM imidazole. Purified protein fractions were pulled and step-dialyzed against a final buffer composition of 10mM Tris-Cl, 50mM NaCl, 1mM DTT and 10% glycerol. Protein concentration was measured with Bradford Assay reagent (Thermo Fisher Scientific, Waltham, MA).

All mutations were generated using In-fusion cloning kit (Clontech, Mountain View, CA) in pET-200- OsADF2 according to manufacturer’s instructions (primers, Supporting information, Table 1). Mutations were confirmed by restriction digestion and/or sequencing. Mutant proteins were purified as described above. His-tag was cleaved in all cases using TAGZyme Kit ( Qiagen, Germantown, MD) unless tag is required for immunoprecipitation.

**Actin polymerization and co-sedimentation assay**

Human platelet G-actin (85% beta and 15% gamma isoforms; Cytoskeleton Inc., Denver, CO) was reconstituted to 0.4 mg/mL in general actin buffer (5 mM Tris-Cl pH 8.0 and 0.2 mM CaCl2) and verified for single band in 12% SDS-PAGE gels. The G-actin was polymerized at RT in a buffer containing 50 mM KCl, 2mM MgCl2 and 1mM ATP for 3 h. The F-actin/actin bundles were separated from the G-actin by centrifugation (40,000 g) at 4 ºC for 3h. The pellet was reconstituted in actin binding buffer (ABB) 10mM Tris, 1mM ATP, 0.2mM DTT, 1mM EGTA, 0.1mM CaCl2 and 2mM MgCl2), and used immediately for binding assays.

SaADF2 and OsADF2 proteins were incubated with bundled/polymerized actin in excess in ABB at RT for 2.5 h and then centrifuged at 25,000 g, 4 ºC for One h. The pellet was dissolved in ABB, precipitated with acetone, and centrifuged. The pellet was then resuspended in Laemmli sample buffer (4% SDS, 20% Glycerol, 120 mM Tris-Cl, pH 6.8) with 0.02% BPB and run in a 12% SDS-PAGE. The gels were scanned in Gel-Doc-it^2^ imager (UVP, Analytik Jena AG, Jena, Germany) and band density was quantified using ImageJ (NIH, Bethesda, MD) and Image Studio Lite (LI-COR Biosciences, Lincoln, NE).

**F-actin depolymerization assay and visualization**

Four μM rabbit muscle 30% pyrene-labeled actin (Cytoskeleton Inc) was polymerized as described by Singh *et al*. (2010). F-actin depolymerization was induced either by diluting samples to a final concentration of 0.4 μM actin or by adding 0.8 μM SaADF2/OsADF2. Pyrene fluorescence was recorded over an h using a Synergy 4 microplate fluorescence reader (BioTek, Winooski, VT) with excitation at 360 nm and emission at 420 nm. The depolymerizing activity was measured either by using pre-polymerized actin or by adding proteins to an actively polymerizing G-actin solution and quantifying the decrease in polymerization relative to polymerizing actin without proteins. The data were acquired in Gen5 1.11 software (Biotek) and analyzed in Microsoft Excel.

Actin filament disassembly and severing by ADF proteins were observed by TIRF (Total Internal Reflection Fluorescence) microscopy, as described by Shekhar and Carlier (2017), with modifications. Briefly, 1mg/ml rabbit muscle skeletal actin (Cytoskeleton Inc, USA) was allowed to polymerize in presence of 1/10th volume 10x polymerization buffer (500mM KCl, 20mM MgCl2, 10mM ATP and 0.1mM EGTA) for one hour in room temperature. Unseeded filaments were then directly labeled on the coverslip in a flow of 1X polymerization buffer containing 70nM Acti-stain 488 Phalloidin. 2.5 μL of filaments were mixed with 8 μM of ADF proteins suspended in assay buffer described earlier and immediately scored. Filaments were observed using Leica DM6000/TIRF system under 63x oil immersion objective and images with CCD camera was captured with a live imager (LA SX). Time-lapse imaging was done at five-second frame intervals spanning 160 seconds. Images were analyzed in ImageJ. All single-filament visualizations have been quantified as described by Chin et al. (2017).

***In vitro* phosphorylation**

*In vitro* phosphorylation was carried out following Allwood et al. (2001) with minor modifications. The expressed Sa/Os/mutant ADF-his fusion proteins and OsCDPK protein was pre-cleared by centrifugation and dephosphorylated with calf intestinal phosphatase(CIP, New England Biolab, Ipswich, MA) prior to the reaction. Phosphorylation reaction mixture contained 60 mM MOPS (pH 8.0), four µM ATP (Amresco, Solon, Ohio), 10 mM mgCl_2_ and 200 µM CaCl_2_. Reaction contained of four µM CDPK and 16 µM Sa/Os/mutant ADF-his fusion proteins. Final volume of reaction was initially maintained at 50 µl, and incubated for 15 minutes at 25ºC without shaking. The volume was then increased to 200 µl with reaction buffer supplemented by 10 µl Protease Inhibitor Cocktail (Sigma-Aldrich Corp.St. Louis, MO) and 20 µM ATP for stability of complex and incubated in ice for two-three hrs. The reaction mixture is then incubated with anti-his antibody (GenWay Biotech, San Diego, CA) for four hrs at 4ºC with mild shaking followed by overnight immunoprecipitation with protein A/G sepharose (Pierce, Waltham, MA). The protein complex was then eluted in low pH and dialyzed. Eluents were immunoblotted with anti-phosphoserine antibody (Abcam, Cambridge, MA). The membrane was CIP-treated prior to blocking with Rabbit serum (Sigma-Aldrich Corp.St. Louis, MO). Membrane was developed following standard protocols, using ECL chemiluminescence kit (Pierce, Waltham, MA).

**Construction of binary vector and development of rice transgenics**

Total RNA was isolated from freshly harvested leaves of greenhouse maintained *S. alterniflora* using the RNeasy plant mini kit (Qiagen, Valencia, CA) and first strand cDNA was synthesized from 1µg RNA using iScript™ cDNA synthesis kit (Bio-Rad, Hercules, CA) as described in Baisakh *et al*. (2012). The complete coding sequence of *SaADF2* was amplified from the first strand cDNA using forward and reverse primers containing *Bgl* II and *Bst* EII restriction endonuclease recognition sites, respectively (Supplementary Table S1). Construction of p35S:*SaADF2* in pCAMBIA1305.1 backbone and its subsequent mobilization into *Agrobacterium tumefaciens* LBA4404 was performed following Baisakh *et al.* (2012).

*Agrobacterium tumefaciens*-mediated transformation of embryogenic callus of rice cultivar ‘Nipponbare’ was performed following Rao *et al.* (2009). One hundred ng of genomic DNA isolated from rice leaf tissues of primary transformants (T_0_) using the CTAB method (Murray and Thompson, 1980) was PCR-analyzed for *SaADF2* using gene-specific primers (Supplementary Table S1). Primary transgenic lines were seed-advanced to T_2_ generation for achieving homozygosity.

**Drought and salinity tolerance assay in rice**

Drought stress was imposed on 50-d-old homozygous progenies of *SaADF2*-OE#23, 38, and 41, *OsADF2*-OE#2, 5, 20, and WT as described in Joshi *et al.* (2014). Three pots, each with single plant of the transgenic lines and WT were used. Volumetric soil moisture content was recorded at 2 d interval after water withdrawal using a portable HH2 Moisture Meter (Delta-T Devices Ltd., England) with Theta probe ML2x.

Three-week-old seedlings of homozygous progenies of *SaADF2*-OE, *OsADF2*-OE and WT rice grown in hydroponics with Yoshida’s nutrient solution (Yoshida *et al.*, 1976) were subjected to salt stress (150 mM NaCl) for a week as described earlier (Baisakh *et al.*, 2012).

**Phenotypic, physiological, biochemical and microscopic analyses**

Control and drought- and salt-stressed plants were observed for common stress-induced phenotypic responses, such as leaf yellowing and drying, rolling, tip burning etc. Physiological traits, such as chlorophyll content, photosynthetic performance ( Fv/Fm), relative water content, stomatal conductance and membrane stability index were measured following the procedures described earlier (Baisakh *et al*., 2012; Joshi *et al*., 2014). Cut-leaf float assay was also performed to determine the chlorophyll bleach as a measure of salt tolerance.

In situ O^2-^ was observed using the nitroblue tetrazolium (NBT) staining method (Jabs *et al*., 1996). In situ H_2_O_2_ was visualized using the 3, 3-diaminobenzidine (DAB) staining method (Thordal-Christensen *et al.*, 1997).

Fully expanded leaves of control and drought (7 DAS) WT and *SaADF2*-OE plants were examined for chloroplast ultrastructure after serial dehydration and cryosectioning with a JEM-1400 Transmission EM optimized for high-contrast imaging as per Marques *et al*. (2016). Scanning electron microscopy of freshly harvested control and drought-stressed leaves was performed with JSM -6610 LV SEM (Baisakh *et al*., 2012).

**(Semi) quantitative reverse transcription PCR**

Total RNA isolated from freshly collected leaf and root tissues of control and stressed *SaADF2*-OE and WT plants was used for first strand cDNA synthesis as described earlier. One µl of 3x diluted first strand cNDA was used for semiquantitative RT-PCR of *SaADF2* and genes, which were overrepresented in the comparative transcriptome analysis (*SaADF2*-OE vs *OsADF2*-OE and WT) and selected from the network analysis using STRING ([www.stringdb.org](http://www.stringdb.org)) and RiceNet v2 (Lee *et al*., 2011) after excluding the hypothetical and ribosomal proteins using gene specific primers (Supplementary Table S1) with rice actin gene 1 (*OsAct*1) as the internal control for template validation.

Quantitative RT-PCR was performed on *SaADF2* and functionally related genes in triplicate following Baisakh *et al*. (2012). Rice elongation factor (*Oself1α*) was used as the reference gene for normalization. The fold-change in expression relative to unstressed control was calculated using the 2^^ddCt^ method (Baisakh *et al*., 2008).

**Leaf protoplast isolation and staining of actin filaments**

Three-day-old seedlings of WT, *SaADF2*- OE, and *OsADF2*-OE were transferred to Yoshida’s nutrient solution without (control) or with mannitol (water stress to ѱ_os_ = -0.3 MPa). After a week, 1-cm-long leaf segments were incubated in PME buffer (100 mM PIPES, 5 mM MgSO_4_, and 10 mM EGTA, pH 6.8) containing 300 mM m-maleimidobenzoyl-N-hydroxysuccinimide ester, 1.5% glycerol, and 0.1% Triton X-100, with gentle agitation for 30 min, followed by rinsing twice with PME. The samples were then fixed in 4% paraformaldehyde for 30 min. Green protoplasts were isolated following Zhang *et al.* (2011). The washed protoplasts were observed for intactness under light microscope. Twenty µl protoplast suspensions were spread on Poly-L-Lysine coated slides and allowed to settle for 5 minutes in a humid chamber. The protoplasts were permeabilized with 3% Triton-X 100 in PBS (pH 7.4) for 1 h and stained with 5IU of Alexa Fluor 488-Phalloidin (Cytoskeleton Inc) in dark for 1 h and mounted in 70% glycerol in PBS. Optical sections in Z-stacks at 1µM interval were taken by LSM700 (Zeiss; 40x/1.3 objective). Alexa-488 Phalloidin was excited with the 488-nm line of an argon laser with the emission set to 550 to 600 nm.

**Bimolecular fluorescence complementation**

*SaADF2* and *OsADF2* cDNAs were fused in frame with the N-terminal of the YFP by cloning into the *Xho* I/*Bam* HI and *Xho* I/*Sal* I site of pA7-NYFP (Pattanaik *et al.*, 2011). The predicted interacting protein cDNAs were cloned in frame with C-terminal of the YFP into the corresponding sites of pA7-CYFP (Pattanaik *et al.*, 2011). Ten µg each of split-YFP constructs were delivered into onion epidermal cells using a Biolistic PDS-1000/He system (Bio-Rad) at 1100 psi following Baisakh *et al*. (2012). Empty split vectors and pYFP were used as negative and positive control, respectively. GFP fluorescence was observed after 22 h under blue light with an Olympus SZH10 GFP-stereomicroscope (excitation 480 nm; emission 535 nm). Digital photographs were taken with a Nikon DXM1200C camera operated with the software ACT-1.

**References:**

**Allwood EG, Smertenko AP, Hussey PJ. (**2001) Phosphorylation of plant actin-depolymerising factor by calmodulin-like domain protein kinase. *FEBS Lett*. **499,** 97-100.

**Chin SM, Jansen S, Goode BL.** 2016. TIRF microscopy analysis of human Cof1, Cof2, and ADF effects on actin filament severing and turnover*. J Mol Biol*. **428,**1604-1616.

**Murray MG, Thompson WF.** 1980. Rapid isolation of high molecular weight plant DNA. *Nucl Acids Res.* **8,** 4321-4325.
